# QIIME 2 Enables Comprehensive End‐to‐End Analysis of Diverse Microbiome Data and Comparative Studies with Publicly Available Data

**DOI:** 10.1002/cpbi.100

**Published:** 2020-04-28

**Authors:** Mehrbod Estaki, Lingjing Jiang, Nicholas A. Bokulich, Daniel McDonald, Antonio González, Tomasz Kosciolek, Cameron Martino, Qiyun Zhu, Amanda Birmingham, Yoshiki Vázquez‐Baeza, Matthew R. Dillon, Evan Bolyen, J. Gregory Caporaso, Rob Knight

**Affiliations:** ^1^ Department of Pediatrics University of California San Diego La Jolla California; ^2^ Division of Biostatistics University of California San Diego La Jolla California; ^3^ Center for Applied Microbiome Science Pathogen and Microbiome Institute Northern Arizona University Flagstaff Arizona; ^4^ Department of Biological Sciences Northern Arizona University Flagstaff Arizona; ^5^ Małopolska Centre of Biotechnology Jagiellonian University Kraków Poland; ^6^ Bioinformatics and Systems Biology Program University of California San Diego La Jolla California; ^7^ Center for Microbiome Innovation University of California San Diego La Jolla California; ^8^ Center for Computational Biology and Bioinformatics University of California San Diego La Jolla California; ^9^ Jacobs School of Engineering University of California San Diego La Jolla California; ^10^ Department of Computer Science and Engineering University of California San Diego La Jolla California; ^11^ Department of Bioengineering University of California San Diego La Jolla California

**Keywords:** bioinformatics, metagenomics, microbiome, QIIME 2, Qiita

## Abstract

QIIME 2 is a completely re‐engineered microbiome bioinformatics platform based on the popular QIIME platform, which it has replaced. QIIME 2 facilitates comprehensive and fully reproducible microbiome data science, improving accessibility to diverse users by adding multiple user interfaces. QIIME 2 can be combined with Qiita, an open‐source web‐based platform, to re‐use available data for meta‐analysis. The following basic protocol describes how to install QIIME 2 on a single computer and analyze microbiome sequence data, from processing of raw DNA sequence reads through generating publishable interactive figures. These interactive figures allow readers of a study to interact with data with the same ease as its authors, advancing microbiome science transparency and reproducibility. We also show how plug‐ins developed by the community to add analysis capabilities can be installed and used with QIIME 2, enhancing various aspects of microbiome analyses—e.g., improving taxonomic classification accuracy. Finally, we illustrate how users can perform meta‐analyses combining different datasets using readily available public data through Qiita. In this tutorial, we analyze a subset of the Early Childhood Antibiotics and the Microbiome (ECAM) study, which tracked the microbiome composition and development of 43 infants in the United States from birth to 2 years of age, identifying microbiome associations with antibiotic exposure, delivery mode, and diet. For more information about QIIME 2, see https://qiime2.org. To troubleshoot or ask questions about QIIME 2 and microbiome analysis, join the active community at https://forum.qiime2.org. © 2020 The Authors.

**Basic Protocol**: Using QIIME 2 with microbiome data

**Support Protocol**: Further microbiome analyses

## INTRODUCTION

This tutorial illustrates the use of QIIME 2 (Bolyen et al., 2019) for processing, analyzing, and visualizing microbiome data. Here we use, as an example, a high‐throughput 16S rRNA gene sequencing study, starting with raw sequences and producing publication‐ready analysis and figures (see Basic Protocol). QIIME 2 can also process other types of microbiome data, including amplicons of other markers such as 18S rRNA, internal transcribed spacers (ITS), and cytochrome oxidase I (COI), shotgun metagenomics, and untargeted metabolomics. We will also show how to combine results from an individual study with data from other studies using the Qiita public database framework (Gonzalez et al., 2018), which can be used to confirm relationships between microbiome and phenotype variables in a new cohort, or to generate hypotheses for future testing.

Typical QIIME 2 analyses can vary in many ways, depending on your experimental and data analysis goals and on how you collected the data. In this tutorial, we use the QIIME 2 command‐line interface, and focus on processing and analyzing a subset of samples from the Early Childhood Antibiotics and the Microbiome (ECAM) study (Bokulich, Chung, et al., 2016). We will start with raw sequence files and use a single analysis pipeline for clarity, but note where alternative methods are possible and why you might want to use them.

### Before Starting

We recommend readers to follow the enhanced live version of this protocol (https://curr-protoc-bioinformatics.qiime2.org) which will be updated frequently to always reflect the newest release of QIIME 2. We also recommend that you read about the core concepts of QIIME 2 (https://docs.qiime2.org/2019.10/concepts/) before starting this tutorial, to familiarize yourself with the platform's main features and concepts, including enhanced visualization methods through QIIME 2 View, decentralized provenance tracking (which ensures reproducible bioinformatics), multiple interfaces (including the Python 3 API and QIIME 2 Studio graphical interface), the plugin architecture (which enables anyone to expand QIIME 2's functionality), and semantic types (which enable QIIME 2 to help users avoid misusing their data). In general, we suggest referring to the QIIME 2 website (https://qiime2.org), which will always be the most up‐to‐date source for information and tutorials on QIIME 2, including newer versions of this tutorial. Questions, suggestions, and general discussion should always be directed to the QIIME 2 Forum (https://forum.qiime2.org). A brief “Glossary of terms” for common QIIME 2 terminology is provided as an Appendix.

## USING QIIME 2 WITH MICROBIOME DATA

### Necessary Resources

#### Hardware

QIIME 2 can be installed on almost any computer system (native installation is available on Mac OS and Linux, or on Windows via a virtual machine). The amount of free disk space and memory that you will need varies dramatically depending on the number of samples and sequences you will analyze and the algorithms you will use to do so. At present, QIIME 2 requires a minimum of 6 to 7 GB for installation, and we recommend a minimum of 4 GB of memory as a starting point for small datasets and 8 GB of memory for most other real‐world datasets. Other types of analyses, such as those using shotgun metagenomics plugins, may require significantly more memory and disk space.

#### Software

An up‐to‐date web browser, such as the latest version of Firefox or Chrome, is needed for visualizations using QIIME 2 View

### Installing QIIME 2

The latest version of QIIME 2, as well as detailed instructions on how to install on various operating systems, can be found at https://docs.qiime2.org. QIIME 2 utilizes a variety of external independent packages, and while we strive to maintain backward compatibility, occasionally changes or updates to these external packages may create compatibility issues with older versions of QIIME 2. To avoid these problems, we recommend always using the most recent version of QIIME 2 available online. The online tutorial will always provide installation instructions for the most up‐to‐date, tested, and stable version of QIIME 2.

#### Troubleshooting

If you encounter any issues with installation, or with any other stage of this tutorial, please get in touch with the QIIME 2 Forum at https://forum.qiime2.org.

The QIIME 2 Forum is the hub of the QIIME 2 user and developer communities. Technical support for users and developers is provided there, free of charge. We try to reply to technical support questions on the forum within 1 to 2 business days (though sometimes we need more time). Getting involved on the QIIME 2 Forum, for example by reading existing posts, answering questions, or sharing resources that you have created such as educational content, is a great way to get involved with QIIME 2. We strive to create an inclusive and welcoming community where we can collaborate to improve microbiome science. We hope you will join us!

### (Re)Activating QIIME 2

If at any point during the analysis the QIIME 2 conda environment is closed or deactivated, QIIME 2 2019.10 can be reactivated by running the following command:

conda activate qiime2‐2019.10

To determine the currently active conda environment, run the following command and look for the line that starts with “active environment”:

conda info

### Using this tutorial

The following protocol was completed using QIIME 2 2019.10 and demonstrates usage with the command line interface (CLI). For users comfortable with Python 3 programming, an application programmer interface (API) version of this protocol is also available at https://github.com/qiime2/paper2/blob/master/notebooks/qiime2‐protocol‐API.ipynb. No additional software is needed for using the API. Jupyter notebooks for both of these protocols are also available at https://github.com/qiime2/paper2/tree/master/notebooks. Finally, an enhanced interactive live version of the CLI protocol is also available at https://curr‐protoc‐bioinformatics.qiime2.org with all intermediate files precomputed. While we strongly encourage users to install QIIME 2 on their own computers and follow along with this tutorial. The enhanced live version provides an alternative for when time and computational resources are limited. Following along with the live version of this protocol enables users to skip any step and instead download the pre‐processed output required for a subsequent step. Additionally, the live version also provides simple “copy to clipboard” buttons for each code block which, unlike copying from a PDF file, retains the original formatting of the code, making it easy to paste into other environments. The enhanced live protocol will also be updated regularly with every new release of QIIME 2, unlike the published version, which will remain static with the 2019.10 version.

### Acquire the data from the ECAM study

In this tutorial, we will be using QIIME 2 to perform cross‐sectional as well as longitudinal analyses of human infant fecal microbiome samples. The samples we will be analyzing are a subset of the ECAM study, which consists of monthly fecal samples collected from children at birth up to 24 months of life, as well as corresponding fecal samples collected from the mothers throughout the same period. The original sequence files from this study are of the V4 region of the 16S rRNA gene that were sequenced across five separate runs (2 × 150 bp) on an Illumina MiSeq machine. To simplify and reduce the computational time required for this tutorial, we have selected the forward reads of a subset of these samples for processing. To follow along with this protocol, create a new directory, then download the raw sequences (∼700 MB) and the corresponding sample metadata file into it:

mkdir qiime2‐ecam‐tutorial
cd qiime2‐ecam‐tutorial
wget ‐O 81253.zip https://qiita.ucsd.edu/public_artifact_download/?artifact_id=81253
unzip 81253.zip
mv mapping_files/81253_mapping_file.txt metadata.tsv

Ignore the warning errors during the unzipping step; this is expected behavior. You can now delete the original zip file 81253.zip to save space.

### Explore sample metadata files

In the previous step, in addition to downloading sequence data, we downloaded a set of researcher‐generated sample metadata. In the context of a microbiome study, sample metadata are any data that describe characteristics of the samples that are being studied, the site they were collected from, and/or how they were collected and processed. In this example, the ECAM study metadata include characteristics like age at the time of collection, birth mode and diet of the child, the type of DNA sequencing, and other information. This is all information that is generally compiled at the time of sample collection, and thus is something the researcher should be working on prior to a QIIME 2 analysis. Suggested standards for the type of study metadata to collect, and how to represent the values, are discussed in detail in MIMARKS and MIxS (Yilmaz et al., 2011). In this article, we also include a Support Protocol on metadata preparation to help users generate quality metadata. In QIIME 2, metadata are most commonly stored as a TSV (i.e., tab‐separated values) file. These files typically have a .tsv or .txt file extension. TSV files are text files used to store data tables, and the format can be read, edited, and written by many types of software, including spreadsheets and databases. Thus, it is usually straightforward to manipulate QIIME 2 sample metadata using the software of your choosing. You can use a spreadsheet program of your choice such as Google Sheets to edit and export your metadata files, but you must be extremely cautious about automatic, and often silent, reformatting of values using these applications. For example, the use of programs like Excel can lead to unwanted reformatting of values, insertion of invisible spaces, or sorting of a table in ways that scramble the connection between sample identifiers and the data. These problems are very common and can lead to incorrect results, including missing statistically significant patterns. See the “Metadata preparation” section in the Support Protocol below for details regarding best practices for creating and maintaining metadata files.

Detailed formatting requirements for QIIME 2 metadata files can be found at https://docs.qiime2.org/2019.10/tutorials/metadata/. Metadata files stored in Google Sheets can be validated using Keemei (Rideout et al., 2016), an open‐source Google Sheets plugin available at https://keemei.qiime2.org. Once Keemei is installed, select Add‐ons > Keemei > Validate QIIME 2 metadata file in Google Sheets to determine whether the metadata file meets the required formatting of QIIME 2.

Open the metadata.tsv file with your software of choice and explore the content. Take note of the column names, as we will be referring to these throughout the protocol. Cual‐ID may be useful for creating sample identifiers, and the Cual‐ID paper (Chase, Bolyen, Rideout, & Caporaso, 2015) provides some recommendations on best practices for creating sample identifiers for data management.

### Importing DNA sequence data into QIIME 2 and creating a visual summary

The next step is to import our DNA sequence data (in this case, from the 16S rRNA gene) into QIIME 2. All data used and generated by QIIME 2, with the exception of metadata, exist as QIIME 2 artifacts and use the .qza file extension. Artifacts are zip files containing data (in the usual formats, such as FASTQ) and QIIME 2−specific metadata describing the various characteristics of the data such as their semantic type, data file format, relevant citations for analysis steps that were performed up to this point, and the QIIME 2 steps that were taken to generate the data (i.e., the data provenance). See the Appendix (Glossary) at the end of this article for additional information.

QIIME 2 allows you to import and export data at many different steps, so that you can export them to other software or try out alternative methods for particular steps. When importing data into QIIME 2, you need to provide detail on what the data are, including the file format and the semantic type. Currently, the most common type of raw data from high‐throughput amplicon sequencing are in FASTQ format. These files may contain single‐end or paired‐end DNA sequence reads, and will be in either multiplexed or demultiplexed format. Multiplexed files typically come as two (or three in the case of paired‐end runs) files consisting of your sequences (forward and/or reverse, often but not always referred to as R1 and R2 reads, respectively) and a separate barcode file (often but not always referred to as the I1 reads). In demultiplexed format, you will have one (or two in the case of paired‐end data) sequence files per sample, as the sequences have already been assigned to their designated sample IDs based on the barcode files. For the demultiplexed format, the sample name will typically be a part of the file name. In this protocol, our sequences are in single‐end demultiplexed FASTQ format produced by Illumina's Casava software. As our data are split across multiple files, to import we will need to provide QIIME 2 with the location of our files and assign them sample IDs; this is done using the manifest file. A manifest file is a user‐created tab‐separated values file with two columns: the first column, sample‐id, holds the name you assign to each of your samples, and the second column, absolute‐filepath, provides the absolute file path leading to your raw sequence files. For example:

**Table:** 

sample‐id absolute‐filepath	
10249.M001.03R	$PWD/demux‐se‐reads/10249.M001.03R.fastq.gz
10249.M001.03SS	$PWD/demux‐se‐reads/10249.M001.03SS.fastq.gz
10249.M001.03V	$PWD/demux‐se‐reads/10249.M001.03V.fastq.gz

Alternatively, your sample metadata file can also be made to double as a manifest file by simply adding the absolute‐filepath column to it; in this protocol we demonstrate the creation and use of a separate manifest file. You can create a manifest file in a variety of ways using your favorite text editor application. Here we use a simple bash script to create ours.

**Figure 1:**
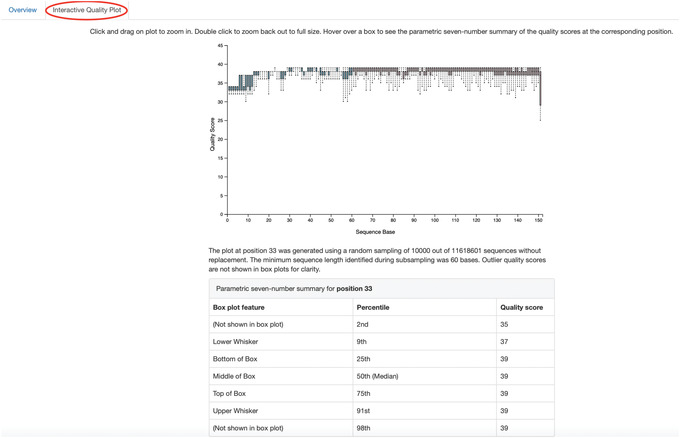
QIIME 2 visualization of the interactive quality plot summarizing the results of demultiplexing sequences.

1 Create the manifest file with the required column headers:
echo ‐e "sample‐id\tabsolute‐filepath" > manifest.tsv
2 Use a loop function to insert the sample names into the sample‐id column, and add the full paths to the sequence files in the absolute‐filepath column:
for f in `ls per_sample_FASTQ/81253/*.gz'; do n=`basename $f';
echo ‐e "12802.${n%.fastq.gz}\t$PWD/$f"; done >> manifest.tsv
3 Use the manifest file to import the sequences into QIIME 2:
qiime tools import \
‐‐input‐path manifest.tsv \
‐‐type `SampleData[SequencesWithQuality]' \
‐‐input‐format SingleEndFastqManifestPhred33V2 \
‐‐output‐path se‐demux.qza
**Alternative Pipeline**
      Your data may not be demultiplexed prior to importing to QIIME 2. Instructions on how to import multiplexed FASTQ files, as well as a variety of other data types, can be found online at https://docs.qiime2.org/2019.10/tutorials/importing/. With multiplexed data, you will also need to demultiplex your sequences prior to the next step. Demultiplexing in QIIME 2 can be performed using either the q2‐demux (https://docs.qiime2.org/2019.10/plugins/available/demux/) plugin which is optimized for data produced using the EMP protocol (Caporaso et al., 2012), or the q2‐cutadapt (https://docs.qiime2.org/2019.10/plugins/available/cutadapt/) plugin (which additionally supports demultiplexing of dual‐index barcodes using cutadapt; Martin, 2011).
  The demultiplexed artifact allows us to create an interactive summary of our sequences. This summary provides information useful for assessing the quality of the DNA sequencing run, including the number of sequences that were obtained per sample and the distribution of sequence quality scores at each position.
4 Create a summary of the demultiplexed artifact:
qiime demux summarize \
‐‐i‐data se‐demux.qza \
‐‐o‐visualization se‐demux.qzv
  You will notice that the output of the summarize action above is a Visualization, with the file extension .qzv. Visualizations are a type of QIIME 2 Result. Like Artifacts, the other type of QIIME 2 Result, they contain information such as metadata, provenance, and relevant citations, but they are outputs that cannot be used as input to other analyses in QIIME 2. Instead, they are intended for human consumption. Visualizations often contain a statistical results table, an interactive figure, one or more static images, or a combination of these. Because they do not need to be used for downstream data analysis in QIIME 2, there is a lot of flexibility in what they can contain. All QIIME 2 Results, including Visualizations and Artifacts, can be viewed by running qiime tools view, or alternatively by loading them with QIIME 2 View (https://view.qiime2.org/). QIIME 2 View does not require QIIME 2 to be installed, making it useful for sharing data with collaborators who do not have QIIME 2 installed. Try visualizing se‐demux.qzv using each of these methods, then use the method you prefer for the rest of this tutorial:
qiime tools view se‐demux.qzv
5 Explore the Visualization results:
  In the first Overview tab, we see a summary of our sequence counts followed by a per‐sample breakdown. If you click on the Interactive Quality plot tab (Fig. 1), you can interact with the sequence quality plot, which shows a boxplot of the quality score distribution for each position in your input sequences. Because it can take a while to compute these distributions from all of your sequence data (often tens of millions of sequences), a subset of your reads are selected randomly (sampled without replacement), and the quality scores of only those sequences are used to generate the box plots. By default, 10,000 sequences are subsampled, but you can control that number with ‐‐p‐n on the demux summarize command. Keep in mind that because of this random subsampling, every time you run demux summarize on the same sequence data, you will obtain slightly different plots.
  Click and drag on the plot to zoom in. When you hover the mouse over a boxplot for a given base position, the boxplot's data are shown in a table below the interactive plot as a parametric seven‐number summary This is a standard summary statistics of a dataset composed of 2nd, 9th, 25th, 50th, 75th, 91st, and 98th percentiles, and can be used as a simple check for assumptions of normality. These values describe the distribution of quality scores at that position in your subsampled sequences. You can click and drag on the plot to zoom in, or double click to zoom back out to full size. These interactive plots can be used to determine if there is a drop in quality at some point in your sequences, which can be useful in choosing truncation and trimming parameters in the next section.

### Sequence quality control and feature table construction

Traditionally, quality control of sequences was performed by trimming and filtering sequences based on their quality scores (Bokulich et al., 2013), followed by clustering them into operational taxonomic units (OTUs) based on a fixed dissimilarity threshold, typically 97% (Rideout et al., 2014). Today, there are better methods for quality control that correct amplicon sequence errors and produce high‐resolution amplicon sequence variants that, unlike OTUs, resolve differences of as little as one nucleotide. These “denoisers” have many advantages over traditional clustering‐based methods, as discussed in Callahan, McMurdie, & Holmes (2017). QIIME 2 currently offers denoising via the DADA2 (q2‐dada2) and Deblur (q2‐deblur) plugins. The inferred ESVs produced by DADA2 are referred to as amplicon sequence variants (ASVs), while those created by Deblur are called sub‐OTUs (sOTUs). In this protocol, we will refer to products of these denoisers, regardless of their method of origin, as *features*. The major differences in the algorithms and motivation for these and other denoising methods are reviewed in Nearing, Douglas, Comeau, & Langille (2018) and Caruso, Song, Asquith, & Karstens (2019). According to these independent evaluations, denoising methods were consistently more successful than clustering methods in identifying true community composition, while only small differences were reported among the denoising methods. We therefore view method selection here as a personal choice that research teams should make. Some practical differences may drive selection of these methods. For instance, DADA2 includes joining of paired‐end reads in its processing workflow, and is therefore simpler to use when paired‐end read joining is desired, while Deblur users must join reads independently prior to denoising using other plugins such as q2‐vsearch’s join‐pairs method (Rognes, Flouri, Nichols, Quince, & Mahé, 2016).

In this tutorial, we will denoise our sequences with q2‐deblur, which uses a pre‐calculated static sequence error profile to associate erroneous sequence reads with the true biological sequence from which they are derived. Unlike DADA2, which creates sequence error profiles on a per‐analysis basis, this allows Deblur to be simultaneously applied across different datasets, reflecting its design motivation to perform meta‐analyses. Additionally, using a pre‐defined error profile generally results in shorter runtimes.

**Figure 2:**
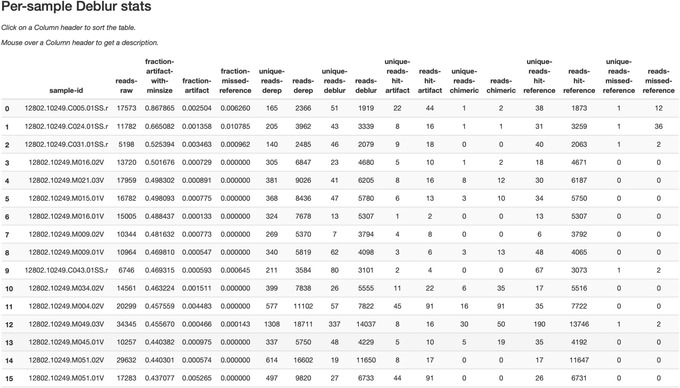
Deblur summary statistics table.

Deblur is applied in two steps:

1 Apply an initial quality filtering process based on quality scores. This method is an implementation of the quality‐filtering approach described by Bokulich et al. (2013):
qiime quality‐filter q‐score \
‐‐i‐demux se‐demux.qza \
‐‐o‐filtered‐sequences demux‐filtered.qza \
‐‐o‐filter‐stats demux‐filter‐stats.qza
2 Apply the Deblur workflow using the denoise‐16S action. This method requires one parameter that is used in quality filtering, ‐‐p‐trim‐length, which truncates the sequences at position *n*. The choice of this parameter is based on the subjective assessment of the quality plots obtained from the previous step. In general, we recommend setting this value to a length where the median quality score begins to drop below 30, or 20 if the overall run quality is too low. One situation where you might deviate from that recommendation is when performing a meta‐analysis across multiple sequencing runs. In this type of meta‐analysis, it is critical that the read lengths be the same for all of the sequencing runs being compared, to avoid introducing a study‐specific bias. In the current example dataset, our quality plot shows high‐quality scores along the full length of our reads; therefore, it is reasonable to truncate our reads at the 150‐bp position:
qiime deblur denoise‐16S \
‐‐i‐demultiplexed‐seqs demux‐filtered.qza \
‐‐p‐trim‐length 150 \
‐‐p‐sample‐stats \
‐‐p‐jobs‐to‐start 4 \
‐‐o‐stats deblur‐stats.qza \
‐‐o‐representative‐sequences rep‐seqs‐deblur.qza \
‐‐o‐table table‐deblur.qza
**Tip!**
      The denoising step is often one of the longest steps in microbiome analysis pipelines. Luckily, both DADA2 and Deblur are parallelizable, meaning you can significantly reduce computation time if your machine has access to multiple cores. To increase the number of cores you wish to designate for this task, use the ‐‐p‐jobs‐to‐start parameter to change the default value of 1 to a value suitable for your machine. As a reminder, if you are following the online version of this protocol, you can skip this step and download the output artifacts, and use those in the following steps.
      Deblur generates three outputs: an artifact with the semantic type FeatureTable[Frequency], which is a table of the counts of each observed feature in each sample, an artifact with the semantic type FeatureData[Sequence], which contains the sequence that defines each feature in the table that will be used later for assigning taxonomy to features and generating a phylogenetic tree, and summary statistics of the Deblur run in a DeblurStats artifact. Each of these artifacts can be visualized to provide important information.
3 Create a visualization summary of the DeblurStats artifact with the command:
qiime deblur visualize‐stats \
‐‐i‐deblur‐stats deblur‐stats.qza \
‐‐o‐visualization deblur‐stats.qzv
  The statistics summary (Fig. 2) provides us with information about what happened to each of the samples during the deblur process. The reads‐raw column gives information on the number of reads presented to the deblur algorithm. Because deblur works by deleting erroneous reads that it detects, the final number of reads is smaller than the starting number. The three columns that follow (fraction‐artifact‐with‐minsize, fraction‐artifact, and fraction‐missed‐reference) summarize the data from other columns in a convenient way. They identify potential problems with the data at an early stage. Fraction‐artifact‐with‐minsize is the fraction of sequences detected as artifactual, including those that fall below the minimum length threshold (specified by the ‐‐p‐trim‐length parameter). Fraction‐artifact is the fraction of raw sequences that were identified as artifactual. Fraction‐missed‐reference is the fraction of post‐deblur sequences that were not recruited by the positive reference database. The subsequent columns provide information about the number of sequences remaining after dereplication (unique‐reads‐derep, reads‐derep), following deblurring (unique‐reads‐deblur, reads‐deblur), number of hits that were recruited to the negative reference database following the deblurring process (unique‐reads‐hit‐artifact, reads‐hit‐artifact), chimeric sequences detected (unique‐reads‐chimeric and reads‐chimeric), sequences that match/miss the positive reference database (unique‐reads‐hit‐reference, reads‐hit‐reference, unique‐reads‐missed‐reference, and reads‐missed‐reference). The number in the reads‐hit‐reference column is the final number of per‐sample sequences present in the table‐deblur.qza QIIME 2 artifact.
**NOTE!**
  The shorthand “artifact” in the per‐sample Deblur statistics denotes artifactual sequences (i.e., those erroneously generated as byproducts of the PCR and DNA sequencing process), not a QIIME 2 artifact (i.e., a valid data product of QIIME 2).

**Figure 3:**
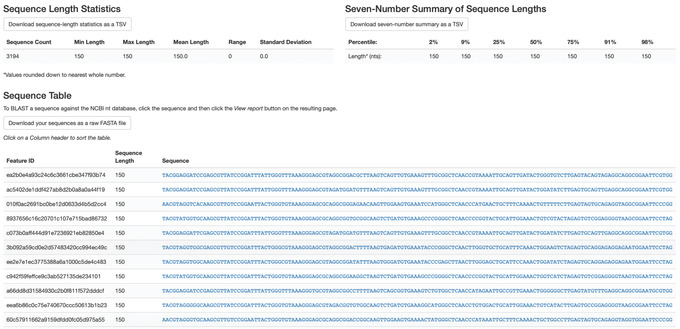
QIIME 2 visualization of deblurred sequences.

4 Visualize the representative sequences by entering:
qiime feature‐table tabulate‐seqs \
‐‐i‐data rep‐seqs‐deblur.qza \
‐‐o‐visualization rep‐seqs‐deblur.qzv
  This Visualization (Fig. 3) will provide statistics and a seven‐number summary of sequence lengths, and, more importantly, show a sequence table that maps feature IDs to sequences, with links that allow you to easily BLAST each sequence against the NCBI nt database. To BLAST a sequence against the NCBI nt database, click the sequence and then click the View report button on the resulting page. This will be useful later in the tutorial, when you want to learn more about specific features that are important in the data set. Note that automated taxonomic classification is performed at a later step, as described below; the NCBI‐BLAST links provided in this Visualization are useful for assessing the taxonomic affiliation and alignment of individual features to the reference database. Results of the “top hits” from a simple BLAST search such as this are known to be poor predictors of the true taxonomic affiliations of these features, especially in cases where the closest reference sequence in the database is not very similar to the sequence that you are using as a query.
**NOTE!**
  By default, QIIME 2 uses MD5 hashing of a feature's full sequence to assign a feature ID. These are the 32‐bit strings of numbers and characters you see in the Feature ID column above. Hashing in q2‐deblur can be disabled by adding the ‐‐p‐no‐hashed‐feature‐ids parameter.

**Figure 4:**
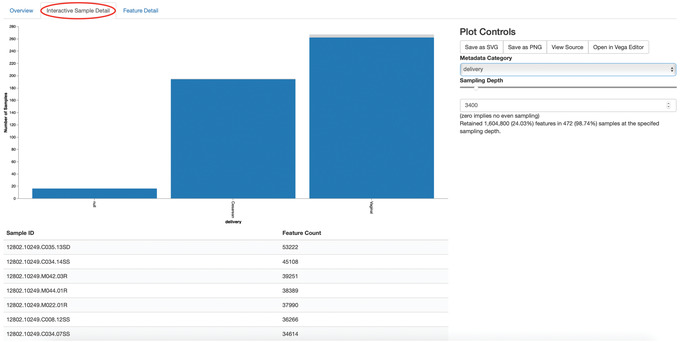
QIIME 2 visualization of interactive sample detail on feature table summaries.

5 Visualize the feature table. Note that in this step we can provide our metadata file, which then adds information about sample groups into the resulting summary output. Adding the metadata is useful for checking that all groups (e.g., a given age or sex of subject) have enough samples and sequences to proceed with analysis. This check is important because variation in the number of sequences per sample, which is typically not fully controllable, often leads to samples being dropped from the analysis because too few reads were obtained from them:
qiime feature‐table summarize \
‐‐i‐table table‐deblur.qza \
‐‐m‐sample‐metadata‐file metadata.tsv \
‐‐o‐visualization table‐deblur.qzv
  The first Overview tab gives information about how many sequences come from each sample, histograms of those distributions, and related summary statistics. The Interactive Sample Detail tab (Fig. 4) shows a bar plot of the number of samples associated with the metadata category of interest, and the feature count in each sample is shown in the table below. Note that you can choose the metadata categories and change sampling depth by dragging the bar or typing in the value. The Feature Detail Detail tab shows the frequency and number of observed samples associated with each feature.
**Alternative Pipeline**
  If traditional OTU clustering methods are desired, QIIME 2 users can perform these using the q2‐vsearch plugin (Rognes et al., 2016) at https://docs.qiime2.org/2019.10/plugins/available/vsearch/. However, we recommend that denoising methods be used prior to clustering in order to utilize the superior quality‐control procedures within these tools.

### Generating a phylogenetic tree

Although microbiome data can be analyzed without a phylogenetic tree, many commonly used diversity analysis methods such as Faith's phylogenetic diversity (Faith, 1992) and UniFrac (Lozupone & Knight, 2005) require one. To use these methods, we must construct a phylogenetic tree that allows us to consider evolutionary relatedness between the DNA sequences.

QIIME 2 offers several methods for reconstructing phylogenetic trees based on features found in your data. These include several variants of traditional alignment‐based methods of building a de novo tree, as well as a fragment‐insertion method that aligns your features against a reference tree. It should be noted that *de novo* trees reconstructed from short sequences result in low‐quality trees because the sequences do not contain enough information to give the correct evolutionary relationships over large evolutionary distances, and thus should be avoided when possible (Janssen et al., 2018). For this tutorial, we will use the fragment‐insertion tree‐building method as described by Janssen et al. (2018) using the sepp action of the q2‐fragment‐insertion plugin, which has been shown to outperform traditional alignment‐based methods with short 16S amplicon data. This method aligns our unknown short fragments to full‐length sequences in a known reference database and then places them onto a fixed tree. Note that this plugin has only been tested and benchmarked on 16S data against the Greengenes reference database (McDonald et al., 2012), so if you are using different data types you should consider the alternative methods mentioned below.

1 Download a backbone tree as the base for our features to be inserted onto. Here, we use the Greengenes (16s rRNA) reference database:
wget \
‐O "sepp‐refs‐gg‐13‐8.qza" \
"https://data.qiime2.org/2019.10/common/sepp‐refs‐gg‐13‐8.qza"
2 Create an insertion tree by entering the following commands:
qiime fragment‐insertion sepp \
‐‐i‐representative‐sequences rep‐seqs‐deblur.qza \
‐‐i‐reference‐database sepp‐refs‐gg‐13‐8.qza \
‐‐p‐threads 4 \
‐‐o‐tree insertion‐tree.qza \
‐‐o‐placements insertion‐placements.qza
  The newly formed insertion‐tree.qza is stored as a rooted phylogenetic tree (of semantic type Phylogeny[Rooted]) and can be used in downstream analysis for phylogenetic diversity computations.
**Tip!**
  Building a tree using SEPP can be computationally demanding and often has longer run times than most steps in a typical microbiome analysis pipeline. The ‐‐p‐threads parameter which, similar to the ‐‐p‐jobs‐to‐start parameter from q2‐deblur, allows this action to be performed in parallel across multiple cores, significantly reduces run time. See the developers’ recommendations with regard to run‐time optimization at https://github.com/qiime2/q2‐fragment‐insertion#expected‐runtimes. As a reminder, if you are following the online version of this protocol, you can skip this step, download the output artifacts, and use those in the following steps.
  Once the insertion tree is created, you must filter your feature table so that it only contains fragments that are in the insertion tree. This step is needed because SEPP might reject the insertion of some fragments, such as erroneous sequences or those that are too distantly related to the reference alignment and phylogeny. Features in your feature table without a corresponding phylogeny will cause diversity computation to fail, because branch lengths cannot be determined for sequences not in the tree.
3 Filter your feature table by running the following:
qiime fragment‐insertion filter‐features \
‐‐i‐table table‐deblur.qza \
‐‐i‐tree insertion‐tree.qza \
‐‐o‐filtered‐table filtered‐table‐deblur.qza \
‐‐o‐removed‐table removed‐table.qza
  This command generates two feature tables: The filtered‐table‐deblur.qza contains only features that are also present in the tree, while the removed‐table.qza contains features not present in the tree. Both of these tables can be visualized as shown in step 5 of the previous section, titled “Sequence quality control and feature table construction.”
**Alternative Pipeline**
  If a traditional de novo phylogenetic tree is desired/required, QIIME 2 offers several methods [FastTree (Price, Dehal, & Arkin, 2010), IQ‐TREE (Nguyen, Schmidt, von Haeseler, & Minh, 2015), and RAxML (Stamatakis, 2014)] to reconstruct these using the q2‐phylogeny plugin (https://docs.qiime2.org/2019.10/plugins/available/phylogeny/). A tree produced by any of these alignment‐based methods can be used with your original feature table without the need for the filtering that SEPP requires. However, if some of your sequences are not 16S rRNA genes, the tree will be incorrect in ways that may severely affect your results.
4 Visualize the phylogenetic tree:
  The phylogenetic tree artifact (semantic type: Phylogeny[Rooted]) produced in this step can be readily visualized using **q2‐empress** (https://github.com/biocore/empress) or iTOL's (Letunic & Bork, 2019) interactive web‐based tool by simply uploading the artifact at https://itol.embl.de/upload.cgi. The underlying tree, in Newick format, can also be easily exported for use in your application of choice (see the “*Exporting QIME 2 data”* section in the Support Protocol, below).

### Taxonomic classification

While sequences derived from denoising methods provide us with the highest possible resolution of our features given our sequencing data, it is usually desirable to know the taxonomic affiliation of the microbes from which sequences were obtained. QIIME 2 provides several methods to predict the most likely taxonomic affiliation of our features through the q2‐feature‐classifier plugin (Bokulich, Kaehler, et al., 2018). These include both alignment‐based consensus methods and Naive Bayes (and other machine‐learning) methods. In this tutorial, we will use a Naive Bayes classifier, which must be trained on taxonomically defined reference sequences covering the target region of interest. Some pre‐trained classifiers are available through the QIIME 2 Data Resources page (https://docs.qiime2.org/2019.10/data‐resources/), and some have been made available by users on the QIIME 2 Community Contributions channel (https://forum.qiime2.org/c/community‐contributions). If a pre‐trained classifier suited for your region of interest or reference database is not available through these resources, you can train your own by following the online tutorial (https://docs.qiime2.org/2019.10/tutorials/feature‐classifier/). In the present protocol, we will train a classifier specific to our data that (optionally) also incorporates environment‐specific taxonomic abundance information to improve species inference. This bespoke method has been shown to improve classification accuracy (Kaehler et al., 2019) when compared to traditional Naive Bayes classifiers, which assume that all species in the reference database are equally likely to be observed in your sample (i.e., that sea‐floor microbes are just as likely to be found in a stool sample as microbes usually associated with stool).

To train a classifier using this bespoke method, we need three files: (1) a set of reference reads, (2) a reference taxonomy, and (3) taxonomic weights. Taxonomic weights can be customized for specific sample types and reference data using the q2‐clawback plugin (Kaehler et al., 2019) (see alternative pipeline recommendation below), or we can obtain pre‐assembled taxonomic weights from the readytowear collection (https://github.com/BenKaehler/readytowear). This collection also contains the reference reads and taxonomies required. The taxonomic weights used in this tutorial have been assembled with 16S rRNA gene sequence data using the Greengenes reference database trimmed to the V4 domain (bound by the 515F/806R primer pair as used in the ECAM study). Here, we will use the pre‐calculated taxonomic weights specific to human stool data. For other sample types, make sure to pick the appropriate weights best fit for your data, and the appropriate sequence reference database; a searchable inventory of available weights is available at https://github.com/BenKaehler/readytowear/blob/master/inventory.tsv.

**Figure 5:**
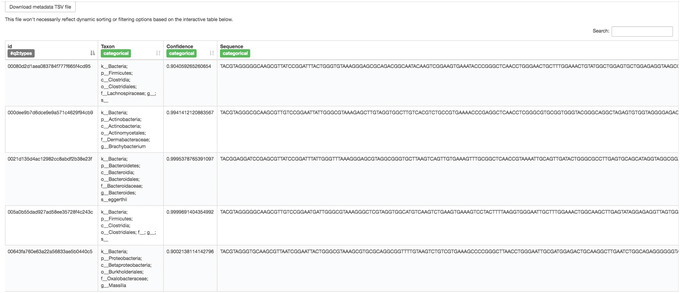
QIIME 2 visualization of classified taxonomy.

1 Start by downloading the three required files from the inventory:
wget
https://github.com/BenKaehler/readytowear/raw/master/data/gg_13_8/515f‐806r/human‐stool.qza
wget
https://github.com/BenKaehler/readytowear/raw/master/data/gg_13_8/515f‐806r/ref‐seqs‐v4.qza
wget
https://github.com/BenKaehler/readytowear/raw/master/data/gg_13_8/515f‐806r/ref‐tax.qza
2 Train a classifier using these files:
qiime feature‐classifier fit‐classifier‐naive‐bayes \
‐‐i‐reference‐reads ref‐seqs‐v4.qza \
‐‐i‐reference‐taxonomy ref‐tax.qza \
‐‐i‐class‐weight human‐stool.qza \
‐‐o‐classifier gg138_v4_human‐stool_classifier.qza
3 Assign taxonomy to our representative sequences using our newly trained classifier:
qiime feature‐classifier classify‐sklearn \
‐‐i‐reads rep‐seqs‐deblur.qza \
‐‐i‐classifier gg138_v4_human‐stool_classifier.qza \
‐‐o‐classification bespoke‐taxonomy.qza
  This new bespoke‐taxonomy.qza data artifact is a FeatureData[Taxonomy] type which can be used as input in any plugins that accept taxonomic assignments.
4 Visualize our taxonomies by entering the following:
qiime metadata tabulate \
‐‐m‐input‐file bespoke‐taxonomy.qza \
‐‐m‐input‐file rep‐seqs‐deblur.qza \
‐‐o‐visualization bespoke‐taxonomy.qzv
  The Visualization (Fig. 5) shows the classified taxonomic name for each feature ID, with additional information on confidence level and sequences. You can reorder the table by clicking the sorting button next to each column name. Recall that the rep‐seqs.qzv Visualization that we created above allows you to easily BLAST the sequence associated with each feature against the NCBI nt database. Using that Visualization and the bespoke‐taxonomy.qzv Visualization created here, you can compare the taxonomic assignments of features of interest with those from BLAST's top hit. Because these methods are only estimates, it is not uncommon to find disagreements between the predicted taxonomies. The results here will generally be more accurate than those received from the simple BLAST search linked from the rep‐seqs.qzv Visualization.
**Alternative Pipeline**
  To assemble your own taxonomic weights for regions not available in the readytowear inventory, follow the detailed instructions at https://forum.qiime2.org/t/using‐q2‐clawback‐to‐assemble‐taxonomic‐weights.

### Filtering data

So far, in addition to our sample metadata, we have obtained a quality‐controlled FeatureTable[Frequency], a Phylogeny[Rooted], and a FeatureData[Taxonomy] artifact. We are now ready to explore our microbial communities and perform various statistical tests. In the following sections, we will explore the microbial communities of our samples from children only, and thus will separate these samples from those of the mothers.

QIIME 2 provides numerous methods to filter your data. These include total feature frequency‐based filtering, identity‐based filtering, metadata‐based filtering, taxonomy‐based filtering, etc. Filtering is performed through the q2‐feature‐table plugin. For a comprehensive list of available filtering methods and examples on how to perform them, visit https://docs.qiime2.org/2019.10/tutorials/filtering/. To separate the child samples, we will use the filter‐samples action to separate samples based on the metadata column “mom_or_child,” where a value of “C” represents a child sample:

qiime feature‐table filter‐samples \
‐‐i‐table filtered‐table‐deblur.qza \
‐‐m‐metadata‐file metadata.tsv \
‐‐p‐where "[mom_or_child]= `C'" \
‐‐o‐filtered‐table child‐table.qza

We now have a new subsetted feature table consisting of child samples only. Let us visualize this new feature table as we did previously:

qiime feature‐table summarize \
‐‐i‐table child‐table.qza \
‐‐m‐sample‐metadata‐file metadata.tsv \
‐‐o‐visualization child‐table.qzv

Load this new Visualization artifact and keep it open, as we will be referring to this in the following section.

### Alpha rarefaction plots

One of the first steps in a typical microbiome analysis pipeline is to evaluate the sampling depth of our samples to determine whether sufficient surveying effort has been achieved. Sampling depth will naturally differ between samples, because the number of sequences generated by current sequencing instruments are neither evenly distributed among samples nor correlated with sample biomass, and, therefore, to avoid bias, must be normalized prior to analysis (e.g., diversity estimates as described below). The methods used for normalization are an active area of research and debate (McMurdie & Holmes, 2014; Weiss et al., 2017). In this section, we will explore how sampling depth impacts alpha diversity estimates (within‐sample richness, discussed in more detail below) using the alpha‐rarefaction action within the q2‐diversity plugin. This Visualizer computes one or more alpha diversity metrics at multiple sampling depths, in steps between 1 (optionally controlled with ‐‐p‐min‐depth) and the value provided, as ‐‐p‐max‐depth. At each sampling‐depth step, 10 rarefied tables will be generated by default, and the diversity metrics will be computed for all samples in the tables. The number of iterations (rarefied tables computed at each sampling depth) can be controlled with ‐‐p‐iterations. Average diversity values will be plotted for each sample at each even sampling depth, and samples can be grouped based on metadata categories in the resulting visualization if sample metadata are provided with the ‐‐m‐metadata‐file parameter:

qiime diversity alpha‐rarefaction \
‐‐i‐table child‐table.qza \
‐‐i‐phylogeny insertion‐tree.qza \
‐‐p‐max‐depth 10000 \
‐‐m‐metadata‐file metadata.tsv \
‐‐o‐visualization child‐alpha‐rarefaction.qzv

Load the child‐alpha‐rarefaction.qzv Visualization.

**Figure 6:**
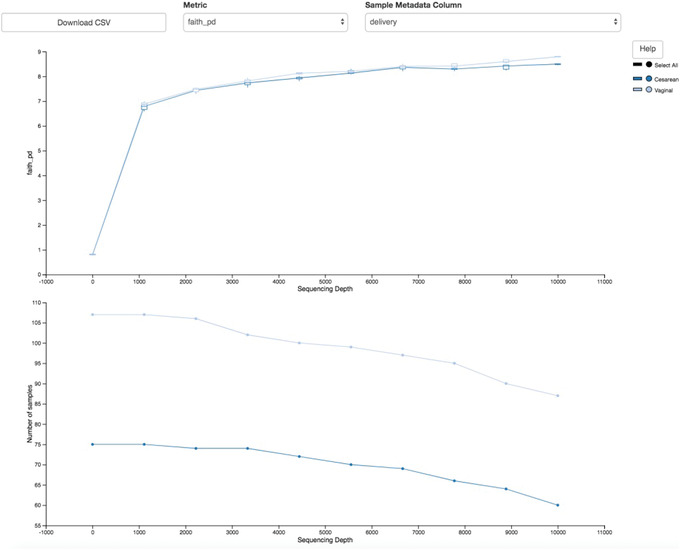
QIIME 2 visualization of alpha diversity rarefaction plots.

The resulting Visualization (Fig. 6) has two plots. The top plot is an alpha rarefaction plot, and is primarily used to determine if the within‐sample diversity has been fully captured. If the lines in the plot appear to “level out” (i.e., approach a slope of zero) at some sampling depth along the *x* axis, this suggests that collecting additional sequences is unlikely to result in any significant changes to our samples’ estimated diversity. If the lines in a plot do not level out, the full diversity of the samples may not have been captured by our sampling efforts, or this could indicate that a lot of sequencing errors remain in the data (which are being mistaken for novel diversity).

The bottom plot in this visualization is important when grouping samples by our metadata categories. It illustrates the number of samples that remain in each group when the feature table is rarefied to each sampling depth. If a given sampling depth “d” is larger than the total frequency of a sample “s” (i.e., the number of sequences that were obtained for sample “s”), it is not possible to compute the diversity metric for sample “s” at sampling depth “d.” If many of the samples in a group have lower total frequencies than “d,” the average diversity presented for that group at “d” in the top plot will be unreliable because it will have been computed on relatively few samples. When grouping samples by metadata, it is therefore essential to look at the bottom plot to ensure that the data presented in the top plot are reliable. Try using the drop‐down menus at the top of the plots to switch between the different calculated diversity metrics and metadata categories.

As mentioned earlier, a normalization method to account for unequal sampling depth across samples in microbiome data is essential to avoid the introduction of bias. One common approach to dealing with this problem is to sample a random subset of sequences without replacement for each sample at a fixed depth (also referred to as rarefying) and discard all remaining samples with total read counts below that threshold. This approach, which is not ideal because it discards a large amount of information (McMurdie & Holmes, 2014), has nonetheless been shown to be useful for many different microbial community analyses that are otherwise dominated by sample‐to‐sample variation in the number of sequences per sample obtained (Weiss et al., 2017). Selecting the depth to which to rarefy samples is a subjective decision motivated by the desire to maximize the rarefying threshold while minimizing loss of samples due to insufficient coverage.

Let us consider our current dataset as an example. In the rarefaction plots above, we can see that there is a natural leveling of our diversity metrics starting at 1000 sequences/sample, with limited additional increases observed beyond 3000 sequences/sample. This should be our target minimum sampling depth. Now let us revisit the child‐table.qzv Visualization from the Filtering data step. Select the Interactive Sample Detail tab from the top left corner, and use the Metadata Category drop‐down menu to select month. Hover over each bar in the plot to see the number of samples included at each month. Now, try moving the Sampling Depth bar on the right starting from the left (zero) to the right. You will see that as the sampling depth increases we begin to rapidly lose samples, as shown by the grayed areas in the bar plot. In this dataset, the time point 0 month is better represented than the subsequent months. We would therefore ideally minimize discarding samples from the other underrepresented months to maintain sufficient statistical power in downstream analyses. Start moving the Sampling Depth bar from zero again; this time stop at the first instance where we begin to see a loss of sample at a month that is not 0. Now scroll down to the bottom of the page. The samples highlighted in red are the would‐be discarded samples at that chosen sampling depth. Here we see that at a depth of exactly 3400 we are able to retain all the samples from months 6, 12, and 24, while still maintaining a minimum depth that will capture the overall signature of the alpha diversity metrics as seen by our rarefaction plots.

#### Alternative Pipeline

Newer methods are actively being developed that circumvent the need for rarefying by taking advantage of the compositional nature of microbiome data; we will show examples of these methods in subsequent sections. However, for some commonly used analysis tasks, no such solution yet exists.

### Basic data exploration and diversity analyses

In the original ECAM study, in addition to monthly sampling, some participants were sampled multiple times in any given month. The exact day at which the samples were collected are recorded in the day_of_life column and again under the month column, with the values in the latter rounded to the nearest month. This rounding process allows us to easily compare samples that were collected at roughly the same month across groups; however, it does introduce artificial replicates, as multiple samples from the same participant will be recorded under the same month. To mitigate the appearance of these false replicates and ensure that samples meet assumptions of independence, we will filter our feature table prior to group tests to include only one sample per subject per month. We have manually identified those samples that would be considered false replicates in the rounding step under the column month_replicate, and will use this to filter our table:

qiime feature‐table filter‐samples \
‐‐i‐table child‐table.qza \
‐‐m‐metadata‐file metadata.tsv \
‐‐p‐where "[month_replicate]= `no'" \
‐‐o‐filtered‐table child‐table‐norep.qza

Create a Visualization summary of this new table as before:

qiime feature‐table summarize \
‐‐i‐table child‐table‐norep.qza \
‐‐m‐sample‐metadata‐file metadata.tsv \
‐‐o‐visualization child‐table‐norep.qzv

We are now ready to explore our microbial communities. One simple method to visualize the taxonomic composition of samples is to visualize them individually as stacked barplots. We can do this easily by providing our feature table, taxonomy assignments, and sample metadata file to the taxa plugin's barplot action.

**Figure 7:**
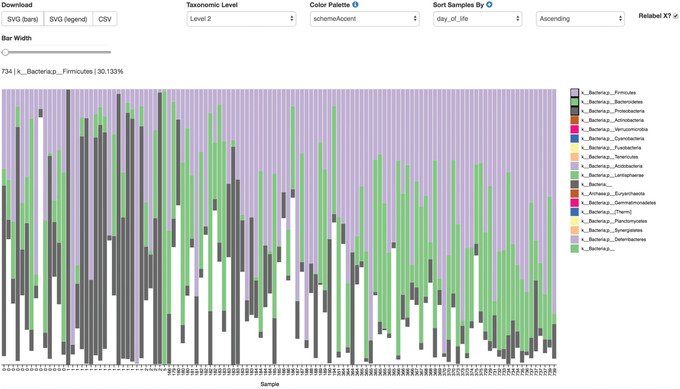
QIIME 2 visualization of taxonomic barplots.

1 Generate the taxonomic barplot by running:
qiime taxa barplot \
‐‐i‐table child‐table‐norep.qza \
‐‐i‐taxonomy bespoke‐taxonomy.qza \
‐‐m‐metadata‐file metadata.tsv \
‐‐o‐visualization child‐bar‐plots.qzv
  This barplot (Fig. 7) shows the relative frequency of features in each sample, where you can choose the taxonomic level to display and sort the samples by a sample metadata category or taxonomic abundance in an ascending or descending order. You can also highlight a specific feature in the barplot by clicking it in the legend. The snapshot above shows a barplot at the phylum level (level 2) where samples were sorted by day. Three phyla were highlighted to show that Proteobacteria (gray) dominate at birth, but by 6 months of age the relative abundance of Bacteroidetes (green) and Firmicutes (purple) make up the majority of the community.
  While barplots can be informative with regard to the composition of our microbial communities, it is difficult to use them to disentangle meaningful signals from noise.
  Many microbial ecology studies use alpha diversity (within‐sample richness and/or evenness) and beta diversity (between‐sample dissimilarity) to reveal patterns in the microbial diversity in a set of samples. QIIME 2's diversity analyses are available through the q2‐diversity plugin, which computes a range of alpha and beta diversity metrics, applies related statistical tests, and generates interactive visualizations. The diversity metrics used in any given study should be based on the overall goals of the experiment. For a list of available diversity metrics in QIIME 2 and a brief description of the motivation behind them, we recommend reviewing the following tutorial: https://forum.qiime2.org/t/alpha‐and‐beta‐diversity‐explanations‐and‐commands.
  In this tutorial, we will utilize the pipeline action core‐metrics‐phylogenetic, which simultaneously rarefies a FeatureTable[Frequency] to a user‐specified depth, computes several commonly used alpha and beta diversity metrics, and generates principal coordinates analysis (PCoA) plots using the EMPeror visualization tool (Vázquez‐Baeza, Pirrung, Gonzalez, & Knight, 2013) for each of the beta diversity metrics. For this tutorial, we will use a sampling depth of 3400 as determined from the previous step.

2 Compute alpha and beta diversity by entering the following commands, minding the ‐‐p‐n‐jobs option if multi‐core usage is desired:
qiime diversity core‐metrics‐phylogenetic \
‐‐i‐table child‐table‐norep.qza \
‐‐i‐phylogeny insertion‐tree.qza \
‐‐p‐sampling‐depth 3400 \
‐‐m‐metadata‐file metadata.tsv \
‐‐p‐n‐jobs 1 \
‐‐output‐dir child‐norep‐core‐metrics‐results
  By default, the following metrics are computed by this pipeline and stored within the child‐core‐metrics‐results directory.

#### Alpha diversity metrics

- Shannon's diversity index (a quantitative measure of community richness; Shannon & Weaver, 1949)
- Observed features (a quantitative measure of community richness, called “observed OTUs” here for historical reasons)
- Evenness (or Pielou's Evenness; a measure of community evenness; Pielou, 1966)
- Faith's Phylogenetic Diversity (a qualitative measure of community richness that incorporates phylogenetic relationships between the features; Faith, 1992); this metric is sometimes referred to as PD_whole_tree, but we discourage the use of that name in favor of Faith's Phylogenetic Diversity or Faith's PD

#### Beta diversity metrics

- Jaccard distance (a qualitative measure of community dissimilarity; (Jaccard, 1908)
- Bray‐Curtis distance (a quantitative measure of community dissimilarity; Sørensen, 1948)
- unweighted UniFrac distance (a qualitative measure of community dissimilarity that incorporates phylogenetic relationships between the features; Lozupone & Knight, 2005); implementation based on Striped UniFrac (McDonald et al., 2018) method
- weighted UniFrac distance (a quantitative measure of community dissimilarity that incorporates phylogenetic relationships between the features; Lozupone, Hamady, Kelley, & Knight, 2007); implementation based on Striped UniFrac (McDonald et al., 2018) method.

After computing the core diversity metrics, we can begin to explore the microbial composition of the samples in the context of their metadata.

### Performing statistical tests on diversity and generating interactive visualizations

#### Alpha diversity

We will first test for associations between our categorical metadata columns and alpha diversity. Alpha diversity asks about the distribution of features within each sample, and once calculated for all samples can be used to test whether the per‐sample diversity differs across different conditions (e.g., samples obtained at different ages). The comparison makes no assumptions about the features that are shared between samples; two samples can have the same alpha diversity and not share any features. The rarefied SampleData[AlphaDiversity] artifact produced in the above step contains univariate, continuous values and can be tested using common non‐parametric statistical test (e.g., Kruskal‐Wallis test) with the following command:

qiime diversity alpha‐group‐significance \
‐‐i‐alpha‐diversity child‐norep‐core‐metrics‐results/shannon_vector.qza \
‐‐m‐metadata‐file metadata.tsv \
‐‐o‐visualization child‐norep‐core‐metrics‐results/shannon‐group‐significance.qzv

Load the newly created shannon‐group‐significance.qzv Visualization.

**Figure 8:**
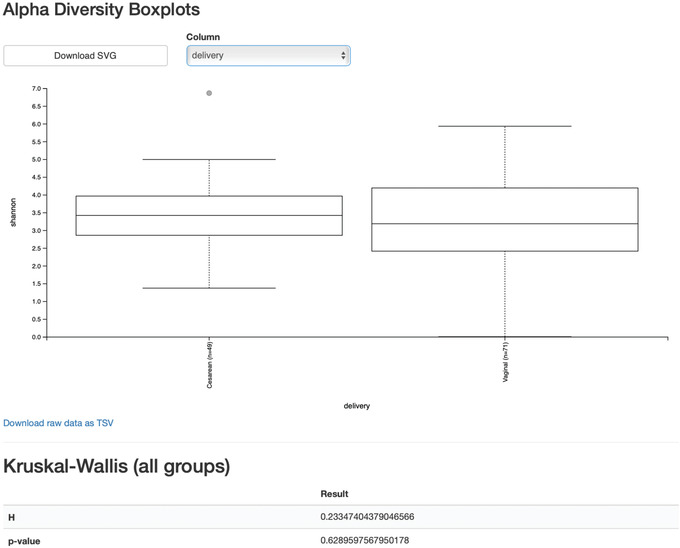
QIIME 2 visualization of alpha diversity comparisons between modes of delivery across all time points.

From the boxplots and Kurskal‐Wallis test results (Fig. 8), it appears that there are no differences between the child samples in terms of Shannon *H* diversity when mode of delivery is considered (*p* value = 0.63). However, exposure to antibiotics appears to be associated with higher diversity (*p* value = 0.026). What are the biological implications?

One important confounding factor here is that we are simultaneously analyzing our samples across all time points and in doing so potentially losing meaningful signals at a particular time point. Importantly, having more than one time point per subject also violates the assumption of the Kurskal‐Wallis test that all samples are independent. More appropriate methods that take into account repeated measurements from the same samples are demonstrated in the longitudinal data analysis section below. It is important to note that QIIME 2 is not able to detect that: you must always be knowledgeable about the assumptions of the statistical tests that you are applying, and whether they are applicable to your data. These types of questions are common on the QIIME 2 Forum, so if you are unsure, start by searching for your question on the forum and posting your own question if you do not find a pre‐existing answer.

So let us re‐analyze our data at the final (month 24) timepoint, by filtering our feature‐table again:

qiime feature‐table filter‐samples \
‐‐i‐table child‐table‐norep.qza \
‐‐m‐metadata‐file metadata.tsv \
‐‐p‐where "[month]= `24'" \
‐‐o‐filtered‐table table‐norep‐C24.qza

Next, we will re‐run the core‐metrics‐phylogenetic pipeline. Visualize the summary of this new table and select a new sampling depth as shown in the previous section. Re‐run core‐metrics‐phylogenetic:

qiime diversity core‐metrics‐phylogenetic \
‐‐i‐table table‐norep‐C24.qza \
‐‐i‐phylogeny insertion‐tree.qza \
‐‐p‐sampling‐depth 3400 \
‐‐m‐metadata‐file metadata.tsv \
‐‐p‐n‐jobs 1 \
‐‐output‐dir norep‐C24‐core‐metrics‐results

And finally, run the alpha‐group‐significance action again:

qiime diversity alpha‐group‐significance \
‐‐i‐alpha‐diversity norep‐C24‐core‐metrics‐results/shannon_vector.qza \
‐‐m‐metadata‐file metadata.tsv \
‐‐o‐visualization norep‐C24‐core‐metrics‐results/shannon‐group‐significance.qzv

Load this new Visualization.

**Figure 9:**
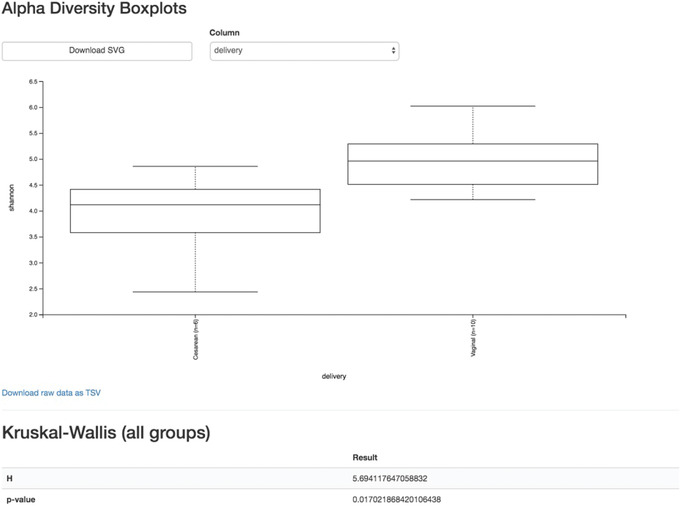
QIIME 2 visualization of alpha diversity at month 24.

We can see now that at month 24, vaginal birth appears to be associated with a higher Shannon value than cesarean birth (*p* value = 0.02, Fig. 9), while antibiotic exposure is no longer associated with differences in Shannon diversity (*p* value = 0.87).

#### Beta diversity

Next, we will compare the structure of the microbiome communities using beta diversity. We start by making a visual inspection of the principal coordinates (PCoA) plots that were generated in the previous step. Load the unweighted_unifrac_emperor.qzv Visualization from the norep‐C24‐core‐metrics‐results folder.

**Figure 10:**
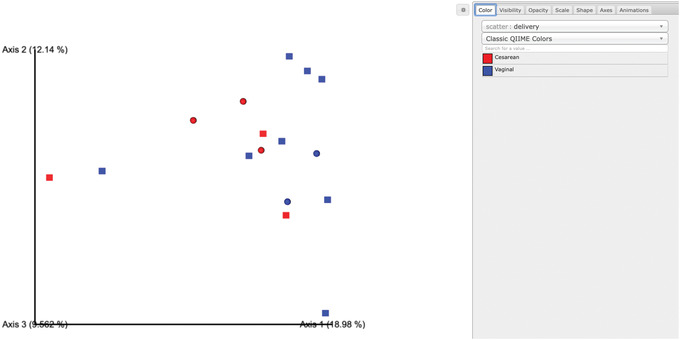
QIIME 2 visualization of PCoA plot using Emperor.

Each dot in the PCoA plot (Fig. 10) represents a sample, and users can color them according to their metadata category of interest and rotate the 3D figure to see whether there is a clear separation in beta diversity driven by these covariates. Moreover, users can customize their figures using existing drop‐down menus by hiding certain samples in Visibility, changing the brightness of dots in Opacity, controlling their size in Scale, choosing different shapes for samples in Shape, modifying the color of axes and background in Axes, and creating a moving picture under the Animations tab.

#### Alternative Pipeline

For longitudinal studies, we have made great use of visualizing temporal variability using animated traces in Emperor. By doing this, you can follow the longitudinal dynamics sample by sample and subject by subject. In order to do so, you need two metadata categories—one to order the samples (Gradient category) and one to group the samples (Trajectory category). For this dataset, we can use animations_gradient as the category that orders the samples, and animations_subject as the category that groups our samples.

The values in animations_gradient represent the age in months. In this category, samples with no longitudinal data are set to 0; note that all values have to be numeric in order for the animation to be displayed. The animations_subject category includes unique identifiers for each subject. Put together, these two categories will result in animated traces on a per‐individual basis.

In Emperor's user interface, go to the Animations tab, select animations_gradient under the Gradient menu, and select animations_subject under the Trajectory menu. Then click play, and you will see animated traces moving on the plot. You can adjust the speed and the radius of the trajectories. To start over, click on the back button. Using the ECAM dataset, we have generated an animation visualizing the temporal trajectories of one vaginally born and one cesarean baby in the 3D PCoA plot. This animation is available at https://tinyurl.com/y7sbzpnd. For more information about animated ordinations, visit Emperor's online tutorial at https://biocore.github.io/emperor/build/html/tutorials/animations.html.

When we color the samples by delivery mode and change the shape of male infants to squares, no obvious clusters are observed. There may be a general trend towards vaginal‐birth children separating from cesarean‐birth samples along Axis 1, which would suggest that the microbial composition of cesarean‐born children is phylogenetically more related within their own groups than that of the vaginal‐birth group. However, given the low sample size in the cesarean group, we are likely underpowered to detect these changes statistically. Nevertheless, we can test our hypothesis using a PERMANOVA, which tests the hypothesis that distances between samples within one group (within‐group distances) differ from the distances to samples in another group (across‐group distances). Other relevant tests in QIIME 2 exist, such as ANOSIM, PERMDISP, or the Mantel test; the choice of test should be carefully considered with regard to the biological question at hand. See Anderson and Walsh (2013) for an overview of these tests. It is also important to note that these tests are useful when testing pre‐existing hypotheses about your data, but cannot be used for testing new hypotheses that were generated by looking at PCoA results. New hypotheses must unfortunately be tested with new, independent data. Here, we perform the PERMANOVA test with the following command:

qiime diversity beta‐group‐significance \
‐‐i‐distance‐matrix norep‐C24‐core‐metrics‐results/unweighted_unifrac_distance_matrix.qza \
‐‐m‐metadata‐file metadata.tsv \
‐‐m‐metadata‐column delivery \
‐‐p‐pairwise \
‐‐o‐visualization norep‐C24‐core‐metrics‐results/uw_unifrac‐delivery‐significance.qzv

Load the Visualization.

**Figure 11:**
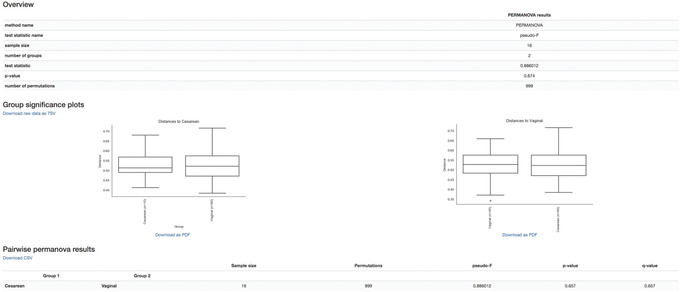
QIIME 2 visualization of PERMANOVA test results.

The overview statistics (Fig. 11) provide us with the parameters used in the PERMANOVA test and the resulting values of test statistic and *p* value. The boxplots (Fig. 9) show the pairwise distance between cesarean and vaginal birth. Lastly, the table (in Fig. 9) summarizes the results from PERMANOVA and gives an additional *q* value (adjusted *p* value for multiple testing). The PERMANOVA test confirms our initial assessment that vaginal‐born microbial communities are not statistically different from cesarean‐born communities in beta diversity (as represented by unweighted UniFrac distances) at month 24 (*p* value = 0.6). These results, however, should be interpreted cautiously given the limited sample size in this dataset. We would conclude that further experiments would be needed to confirm our findings.

#### Alternative Pipeline

The beta diversity analysis above was carried on a rarefied subset of our data. An alternative method that does not require rarefying is offered through the external q2‐deicode plugin (https://library.qiime2.org/plugins/deicode). DEICODE is a form of Aitchison Distance that is robust with respect to compositional data with high levels of sparsity (Martino et al., 2019). This plugin can be used to generate a beta diversity ordination artifact that can easily be utilized with the existing architecture in QIIME 2 such as visualization with q2‐emperor and hypothesis testing with the beta‐group‐significance as above.

### Longitudinal data analysis

When microbial data are collected at different timepoints, it is useful to examine dynamic changes in the microbial communities (longitudinal analysis). This section is devoted to longitudinal microbiome analysis using the q2‐longitudinal plugin (Bokulich, Dillon, Zhang, et al., 2018). This plugin can perform a number of analyses such as: visualization using volatility plots, testing temporal trends in alpha and beta diversities, using linear mixed‐effects models to test for changes in diversity metrics or individual features with regard to metadata categories of interest, and more. A comprehensive list of available methods and instructions on how to perform them are available in the online tutorial: https://docs.qiime2.org/2019.10/tutorials/longitudinal/. Here we will demonstrate some of these methods.

#### Linear mixed effects (LME) models

In a previous section, we determined that Shannon diversity was significantly lower in cesarean‐born children at 24 months of age than in vaginal‐born children. But what about the change in Shannon diversity throughout the 24 months? LME models enable us to test the relationship between a single response variable (i.e., Shannon metric) and one or more independent variables (e.g., delivery mode, diet), where observations are made across dependent samples, e.g., in repeated‐measures sampling experiments. LME models can also account for a random effect (e.g., individuals, sampling times) variable. Here we will use the linear‐mixed‐effects action, which requires the following inputs: the diversity metric of choice calculated for all samples across 24 months (in the child‐core‐metrics‐results folder), the metric name, our sample metadata file, a comma‐separated list of covariates to include in the model, the random‐effect variable (day_of_life), the column name from the metadata file containing the numeric state (i.e., day_of_life), as well as the column name from the metadata file containing the individuals’ ID names to track through time. Unlike the group‐significant tests in the previous steps, LME models can handle continuous variables; therefore, we will utilize our full dataset by calling on the day_of_life column instead of month. We will need to calculate our diversity metrics again on the full dataset as it was before replicates were removed:

qiime diversity core‐metrics‐phylogenetic \
‐‐i‐table child‐table.qza \
‐‐i‐phylogeny insertion‐tree.qza \
‐‐p‐sampling‐depth 3400 \
‐‐m‐metadata‐file metadata.tsv \
‐‐p‐n‐jobs 1 \
‐‐output‐dir child‐core‐metrics‐results

To demonstrate how covariates can be included in an LME model, here we will test the effects of delivery method and diet (predominantly breast‐fed versus predominantly formula‐fed during the first 3 months of life) simultaneously using the following:

qiime longitudinal linear‐mixed‐effects \
‐‐m‐metadata‐file metadata.tsv \
‐‐m‐metadata‐file \ child‐core‐metrics‐results/shannon_vector.qza \
‐‐p‐metric shannon \
‐‐p‐random‐effects day_of_life \
‐‐p‐group‐columns delivery,diet \
‐‐p‐state‐column day_of_life \
‐‐p‐individual‐id‐column host_subject_id \
‐‐o‐visualization lme‐shannon.qzv

**Figure 12:**
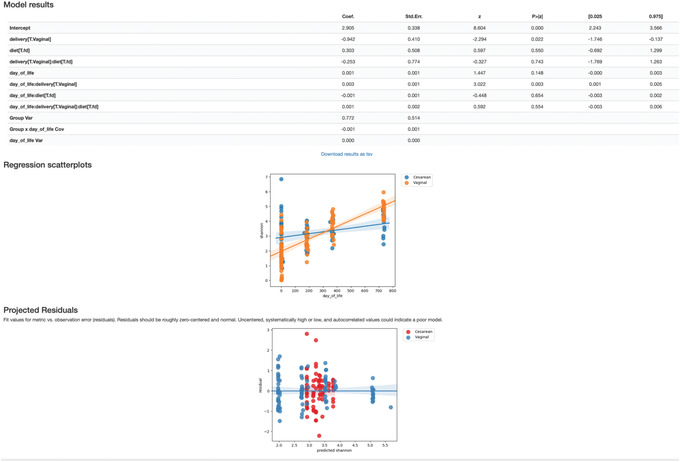
QIIME 2 visualization of Linear Mixed Effects Model results.

In this Visualization (Fig. 12), the model results provide all the outputs from the LME model, where we see a significant birth mode effect in Shannon diversity over time (*p* value = 0.02), while the diet has no bearing in Shannon diversity across time (*p* value = 0.55). The regression scatterplots (top) overlap the predicted group mean trajectories on the observed data (dots), and the projected residuals plot (bottom) can help users to check the validity of an LME model. For more details, see https://docs.qiime2.org/2019.10/tutorials/longitudinal/.

#### Volatility visualization

The volatility visualizer generates interactive line plots that allow us to assess how volatile a dependent variable is over a continuous, independent variable (e.g., time) in one or more groups. Multiple metadata files (including alpha and beta diversity) and feature tables can be used as input, and in the interactive visualization we can select different dependent variables to plot on the *y* axis. Here we examine how variance in Shannon diversity changes across time in our cohort, both in groups of samples (interactively selected) and in individual subjects.

The volatility plot can be generated by running:

qiime longitudinal volatility \
‐‐m‐metadata‐file metadata.tsv \
‐‐m‐metadata‐file \ child‐core‐metrics‐results/shannon_vector.qza \
‐‐p‐default‐metric shannon \
‐‐p‐default‐group‐column delivery \
‐‐p‐state‐column month \
‐‐p‐individual‐id‐column host_subject_id \
‐‐o‐visualization shannon‐volatility.qzv

**Figure 13:**
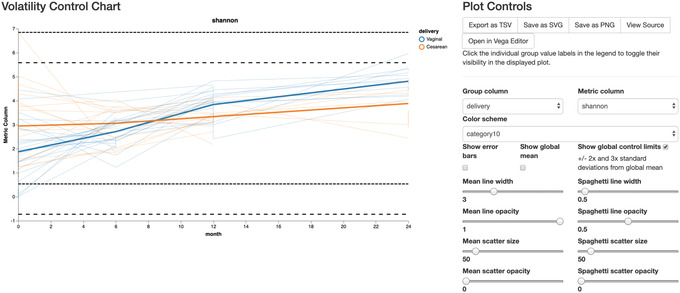
QIIME 2 visualization of volatility analysis.

The volatility plot (Fig. 13) shows the mean curve of each group in the selected group column on top of individual trajectories over time. This plot can be useful in identifying outliers qualitatively, by turning on show global control limits to show ± 2× and 3× standard deviation lines from global mean. Observations above those global control limits are suspected to be outliers. In this analysis, we see high variance at time zero, while they become more similar by month 8, and by month 24, vaginally born children appear to be higher than cesarean‐born (as expected).

#### Differential abundance testing

So far, we have analyzed our data using a variety of approaches employing various diversity metrics and between‐sample distances that are useful in comparing our communities in a broad approach. Now, we want to identify individual taxa whose relative abundances are significantly different across groups. Differential abundance testing in microbiome analysis is an active area of research (see the “compositional data analysis” section in the Support Protocol for more details). Two QIIME 2 plugins that can be used for this are q2‐songbird (Morton et al., 2019) and q2‐composition. In this section, we will use the ANCOM test in the q2‐composition plugin to identify differential abundant features between vaginal‐ and cesarean‐born children. Moreover, we will use q2‐songbird to perform a similar task, but with the additional adjustment for potential confounders.

#### ANCOM

As with any bioinformatics method, you should be aware of the assumptions and limitations of ANCOM before using it. For example, ANCOM assumes that few (less than ∼25%) features differ between groups. If you expect that more features differ between your groups, you should not use ANCOM because it will be more error‐prone (an increase in both Type I and II errors is possible). We recommend reading the ANCOM paper (Mandal et al., 2015) before using this method. For the simplicity of the analysis, we will focus on identifying differential abundant features in children born with different birth modes at month 6 only. We have selected 6 months because this time point contains the highest number of samples (after baseline time 0), which greatly increases the power of our analysis.

**Figure 14:**
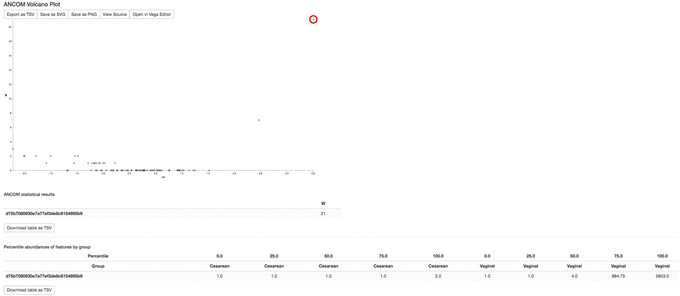
QIIME 2 visualization of ANCOM results (identified features circled in red).

1 Create a new feature‐table that contains only samples from children at 6 months:
qiime feature‐table filter‐samples \
‐‐i‐table child‐table‐norep.qza \
‐‐m‐metadata‐file metadata.tsv \
‐‐p‐where "[month]=`6'" \
‐‐o‐filtered‐table table‐norep‐C6.qza
  When performing differential abundance testing, it is generally a good idea to filter out features that have very low abundances across your dataset, as well those that are present in only a few samples. These features tend to add noise to the results, so we will remove them. Here we use the filter‐features action to filter out features appearing in less than ∼10% of our samples (min 5 of 43 samples) and those that have a total frequency less than 20 counts across all samples.
2 Filter out features with the following commands:
qiime feature‐table filter‐features \
‐‐i‐table table‐norep‐C6.qza \
‐‐p‐min‐samples 5 \
‐‐p‐min‐frequency 20 \
‐‐o‐filtered‐table filtered‐table‐C6.qza
  Because ANCOM operates on relative abundance data, it requires as input a feature table of type FeatureTable[Composition]; it also cannot tolerate frequencies of zero. To resolve both of these requirements, we will use the add‐pseudocount action to simultaneously apply relative abundance transformation and add a pseudocount of 1 to all of our counts.
3 Add pseudocount to the filtered feature table:
qiime composition add‐pseudocount \
‐‐i‐table filtered‐table‐C6.qza \
‐‐o‐composition‐table comp‐table‐C6.qza
4 Run ANCOM to determine which features differ in relative abundance across the different birth modes:
qiime composition ancom \
‐‐i‐table comp‐table‐C6.qza \
‐‐m‐metadata‐file metadata.tsv \
‐‐m‐metadata‐column delivery \
‐‐o‐visualization ancom‐C6‐delivery.qzv
  The Visualization of ANCOM results (Fig. 14) first shows a volcano plot, where the *x* axis summarizes the effect size difference of the given features between interested metadata categories (delivery modes in our case) and the *y* axis is the strength of the ANCOM test statistic W. As ANCOM is essentially running pairwise tests, the W value is a count of the number of sub‐hypotheses that have passed for a given feature. Hence, the differentially abundant features will be those ASVs with high values on both the *x* and *y* axis, in other words, points that are close to the top right or left corners (in the figures for this tutorial, the one identified feature has been highlighted with a red circle). The identified features are summarized underneath the ANCOM statistical results section. Lastly, the percentile abundance table shows the number of sequences assigned to each identified feature in how many of the samples. Regarding the identified feature in our analysis, of the samples in the cesarean group, in the sample with the lowest count of sequences assigned to detected feature, one sequence was observed that was ultimately assigned to this feature. Then, in 75% of the samples in the cesarean group, 1 or fewer sequences were observed that were ultimately assigned to this feature (recall that adding the pseudocount ensures that every sample will appear to have at least 1 count of every feature). However, in 75% of the samples in the Vaginal group, 884.75 or fewer sequences were observed that were ultimately assigned to this feature. This percentile abundance table suggests that the detected feature is higher in vaginally born than cesarean‐born babies.
  The ANCOM test has identified 1 feature that differs significantly by birth mode. To identify which taxa this feature corresponds to, we can load our bespoke‐taxonomy.qzv artifact made in step 4 of the “Taxononomic classification” section and look up the feature ID in the search‐bar at the top.
  This identified feature and its corresponding taxonomic assignment are as follows:
  Feature with higher abundance in vaginal‐born children:
d75b7080930e7a77ef3de8c6154895b9 ‐>
k__Bacteria; p__Actinobacteria; c__Actinobacteria;
o__Bifidobacteriales; f__Bifidobacteriaceae;
g__Bifidobacterium; s__
  Perhaps not surprisingly, these results echo findings from the original ECAM paper (Bokulich, Chung, et al., 2016) encompassing the full dataset.

#### Songbird

Songbird (Morton et al., 2019) can be used to identify differentially abundant features while accounting for confounding variables in the data. This is a multinomial regression designed for compositional microbiome data (in technical terms, it is an L2 regularized multinomial regression that avoids overfitting by using the sum of squares of all feature weights as penalty term to the loss function, as in Ridge regression). Here, we control for confounding variables such as antibiotic exposure and infants’ diet and sex when identifying features that are significantly different between babies born vaginally versus through C‐section.

1 Install the songbird qiime2 plugin (https://github.com/biocore/songbird) in your QIIME 2 environment and make a folder to store the songbird results by running:
conda install songbird ‐c conda‐forge
mkdir songbird‐results
2 Run songbird with the following command:
qiime songbird multinomial \
‐‐i‐table table‐norep‐C6.qza \
‐‐m‐metadata‐file metadata.tsv \
‐‐p‐formula "delivery+abx_exposure+diet+sex" \
‐‐p‐epochs 10000 \
‐‐p‐differential‐prior 0.5 \
‐‐o‐differentials songbird‐results/differentials6monthControlled.qza \
‐‐o‐regression‐stats songbird‐results/regression‐stats6monthControlled.qza \
‐‐o‐regression‐biplot songbird‐results/regression‐biplot6monthControlled.qza
  Note that users can adjust their model parameters and validate their fitted models by using the existing model diagnostic tools in songbird, such as plotting graphs of prediction accuracy and visualizing convergence summary.
3 Examine the estimated coefficients for each feature by running:
qiime tools export \
‐‐input‐path songbird‐results/differentials6monthControlled.qza \
‐‐output‐path songbird‐results/exported‐differentials6monthControlled
  Based on the estimated coefficients for delivery[T.Vaginal] in the output of regression stats, we consider the features with the positive coefficients to be differential relative to negative coefficients in vaginal‐born infants versus cesareans, and vice versa. There is no clear cutoff in songbird on the value of coefficients to assist in choosing of number of features, but since there are few features with coefficients higher than 2.5 or lower than ‐2.5, we use this threshold as our cut‐off for regression coefficients and thus identify five vaginally born‐associated and four C‐section born‐associated features, as shown below:
  Features with higher differential ranking in vaginal‐born children (listed from strongest to weakest):
d75b7080930e7a77ef3de8c6154895b9 ‐>
k__Bacteria; p__Actinobacteria; c__Actinobacteria;
o__Bifidobacteriales; f__Bifidobacteriaceae; g__Bifidobacterium; s__
2a99ec1157a90661db7ff643b82f1914 ‐>
k__Bacteria; p__Bacteroidetes; c__Bacteroidia; o__Bacteroidales;
f__Bacteroidaceae; g__Bacteroides; s__fragilis
c162a4f3943238810eba8a25f0563cca ‐>
k__Bacteria; p__Bacteroidetes; c__Bacteroidia; o__Bacteroidales;
f__Bacteroidaceae; g__Bacteroides; s__ovatus
c4f9ef34bd2919511069f409c25de6f1 ‐>
k__Bacteria; p__Bacteroidetes; c__Bacteroidia; o__Bacteroidales;
f__Bacteroidaceae; g__Bacteroides; s__
  Features with higher differential ranking in caesarian‐born children (listed from strongest to weakest):
1ad289cd8f44e109fd95de0382c5b252 ‐>
k__Bacteria; p__Firmicutes; c__Clostridia; o__Clostridiales;
f__Lachnospiraceae; g__Clostridium; s__hathewayi
C18afe570abfe82d2f746ecc6e291bab ‐>
k__Bacteria; p__Proteobacteria; c__Gammaproteobacteria;
o__Enterobacteriales; f__Enterobacteriaceae; g__Klebsiella; s__
bca0b81a0b8d59e90c25a323c2f62f31 ‐>
k__Bacteria; p__Firmicutes; c__Clostridia;
o__Clostridiales; f__Clostridiaceae; g__Clostridium; s__perfringens

### Meta‐analysis through the Qiita database using redbiom

After identifying differentially abundant features using ANCOM or songbird, users can search through available samples in Qiita (Gonzalez et al., 2018) using redbiom (McDonald et al., 2019) to see the characteristics of samples. This type of analysis can be used to examine what environments a particular feature was previously observed in. In addition, the FeatureTable[Frequency] data for the samples that contain a feature of interest can be extracted for further analysis. A detailed tutorial can be found on the QIIME 2 Forum (https://forum.qiime2.org/t/querying‐for‐public‐microbiome‐data‐in‐qiita‐using‐redbiom/4653). Here, we will search an individual differentially abundant feature to see whether that feature appears enriched in different infants by birth mode. Note that the exact numbers and results shown below may change over time as more samples get indexed by redbiom.

To use redbiom, we first need to install the package using conda:

conda install ‐c conda‐forge redbiom

In redbiom, the data are partitioned by technical and processing parameters to help improve the comparability of the contained data. Before we search for features, we need to decide the context to search within. The redbiom summarize contexts command provides information about the names of the contexts and the number of samples and features indexed. The context names themselves describe the processing parameters used:

redbiom summarize contexts

This produces a long output, as there are several sequencing technologies, sequence trim lengths, a few different variable regions, and multiple feature assessment methods represented. The first five lines of this output are shown below, which provide the context name, the number of samples in the context, the number of unique features, and a succinct description of the bioinformatic processing performed:

ContextName  SamplesWithData  FeaturesWithData  Description

Pick_closed‐reference_OTUs‐Greengenes‐Illumina‐16S‐V4‐125nt‐65468f  16622  40899  Pick closed‐reference OTUs (reference‐seq: |databases|gg|13_8|rep_set|97_otus.fasta) | Trimming (length: 125)

Deblur‐Illumina‐16S‐V4‐150nt‐780653  127413  7299964  Deblur (Reference phylogeny for SEPP: Greengenes_13.8, BIOM: reference‐hit.biom) | Trimming (length: 150)

Pick_closed‐reference_OTUs‐Greengenes‐LS454‐16S‐V4‐41ebc6  7326  27248  Pick closed‐reference OTUs (reference‐seq: |databases|gg|13_8|rep_set|97_otus.fasta) | Split libraries

Pick_closed‐reference_OTUs‐Greengenes‐LS454‐16S‐V4‐100nt‐a243a1  7434  29507  Pick closed‐reference OTUs (reference‐seq: |databases|gg|13_8|rep_set|97_otus.fasta) | Trimming (length: 100)

Deblur‐Illumina‐16S‐V4‐125nt‐3aae8b  15064  378537  Deblur (Reference phylogeny for SEPP: Greengenes_13.8, BIOM: reference‐hit.biom) | Trimming (length: 125)

For the analysis here, we are going to use the Deblur‐Illumina‐16S‐V4‐150nt‐780653 context; this context is composed of samples which sequenced the 16S V4 region, are all 150 nucleotides in length, and were processed with Deblur. The context contains 127,413 samples spanning over 7.2 million unique features, representing hundreds of publicly available studies in Qiita.

Next, we will take the DNA sequence corresponding to our feature of interest d75b7080930e7a77ef3de8c6154895b9, identify samples within the context in which the sequence was observed, and save the output into a file called observed_samples.txt. Note that feature hashes cannot presently be used for search; use the bespoke‐taxonomy.qzv Visualization to locate its corresponding DNA sequences:

redbiom search features ‐‐context Deblur‐Illumina‐16S‐V4‐150nt‐
780653\
TACGTAGGGTGCAAGCGTTATCCGGAATTATTGGGCGTAAAGGGCTCGTAGGCGGTTCGTCGCGTCCGGTGTGAAAGTCCATCGCTTAACGGTGGATCTGCGCCGGGTACGGGCGGGCTGGAGTGCGGTAGGGGAGACTGGAATTCCCGG > observed_samples.txt

If we examine the observed_samples.txt file, we will see that over 17,000 samples contain this particular feature. These samples are part of 137 different studies in Qiita. We can now begin to explore what is known about the samples. A major challenge for meta‐analysis, though, is having common metadata categories across studies.

First, as a sanity check, we will search against only those samples that record the Earth Microbiome Project Ontology (Thompson et al., 2017). The EMPO_3 level describes basic environmental information about a sample. Only samples that describe an entry in their metadata for empo_3 will be obtained:

redbiom summarize samples \
‐‐category empo_3 \
‐‐from observed_samples.txt

What we can see from this output is that (as expected) the feature is primarily observed in samples associated with the animal distal gut:

**Table:** 

Animal distal gut	7124
Animal surface	331
Surface (non‐saline)	204
Sterile water blank	102
Animal secretion	91
animal distal gut	68
Animal corpus	58
Water (non‐saline)	15
Plant corpus	13
Animal proximal gut	12
Aerosol (non‐saline)	9
Single strain	6
Water (saline)	6
Soil (non‐saline)	6
not provided	2
Sediment (saline)	2
Surface (saline)	1
Total samples	8050

Now, let us search this feature against only those samples that correspond to infants. For that, we will need to select the set of samples that correspond to a particular criterion. In this case, we will filter to include only samples associated with individuals under the age of 3. The two metadata categories we will use are host_age and ages, both of which are common labels in Qiita that correspond to an individual's recorded age. In addition, we will explicitly omit the ECAM study from our qiita search, as our dataset was drawn from this study:

redbiom select samples‐from‐metadata \
‐‐context Deblur‐Illumina‐16S‐V4‐150nt‐780653 \
‐‐from observed_samples.txt "where (host_age < 3 or age < 3) and qiita_study_id != 10249" > infant_samples.txt

We can then summarize the metadata of these infant samples. In order to do so, we need to determine what metadata category to summarize over. So let us search Qiita for all metadata categories (not shown below) that contain the word ‘birth’ in the name, pick a few that seem plausible, and summarize them:

redbiom search metadata \
‐‐categories birth
redbiom summarize metadata birth_method birth_mode

We can see that birth_mode is represented by thousands of samples:

**Table:** 

birth_method	72
birth_mode	2176

So, let us use that metadata category:

redbiom summarize samples \
‐‐category birth_mode \
‐‐from infant_samples.txt

From this summary, it appears our feature of interest is present in many more samples associated with a vaginal birth than cesarean section:

**Table:** 

Vaginal	38
Cesarea	16
Vag	3
CSseed	1
Total samples	58

It is important to note, however, that these findings may be confounded by the possibility that there may be more representations of vaginal birth samples in Qiita. However, a summary of that metadata category across all of Qiita can be performed easily:

redbiom summarize metadata‐category \
‐‐counter \
‐‐category birth_mode

This suggests the variable is not extremely unbalanced between C‐section and vaginal births, and that actually more of the samples are associated with C‐sections.

**Table:** 

Category	value count
Cesarea	47
Vaginal	135
CSseed	335
Vag	689
CS	970

Last, we can see the studies these samples were observed in by summarizing over the qiita_study_id category:

redbiom summarize samples \
‐‐category qiita_study_id \
‐‐from infant_samples.txt

We see that nine different Qiita studies are represented by the infant samples:

**Table:** 

10581	54
10918	30
11076	19
1064	15
11358	10
11947	10
2010	4
10512	3
11284	1
Total samples	146

Further exploration of these samples can be performed, such as extracting the samples and integrating them directly in a meta‐analysis (see redbiom fetch to obtain feature tables and sample metadata).

## FURTHER MICROBIOME ANALYSES

The following sections are offered as stand‐alone additional support for further microbiome analyses and do not rely on the ECAM dataset used in previous sections.

### Exporting QIIME 2 data

Occasionally, the raw data within QIIME 2 artifacts may be required for use in other applications that cannot read these file types. QIIME 2 artifacts are simple zip files, and so their content can be extracted using any unzipping software. They can also be extracted readily using the qiime tools extract plugin, which extracts the raw data as well as QIIME 2's metadata about that artifact—including for example the artifact's provenance—into the output directory in plain‐text format. The extracted files will be placed in a new directory whose name is the artifact's universally unique identifier (UUID). Alternatively, when only the raw data are desired without the metadata, qiime tools export can be used. When exporting an artifact, only the data files will be placed in the output directory. For example, a user may be interested in visualizing a phylogenetic tree using a package in R. To obtain the raw tree file (in Newick format), simply run:

qiime tools export \
‐‐input‐path insertion‐tree.qza \
‐‐output‐path extracted‐insertion‐tree

### Analysis of shotgun metagenomic data

Whole‐metagenome shotgun (WMS) sequencing explores the totality of genomes in the microbial community. Compared to amplicon‐based analyses, it provides higher taxonomic resolution (typically beyond the genus level), direct observation of functional genes, and further information on the genome organization. Although assembly into draft genomes usually demands high sequencing depth, which is expensive, investigation of the microbial community can be as affordable as amplicon sequencing, hence enabling survey of larger quantity of samples. It has been demonstrated that “shallow” shotgun sequencing (0.5 million sequences per sample) delivers nearly equal insights into the community's taxonomic composition compared to sequencing with 100 times as much depth (Hillmann et al., 2018)—although functional profiles are not nearly as accurate in shallow shotgun sequencing. Therefore, experimental design and budget arrangements should be made based on the goals of the study.

Two plugins that are dedicated to shotgun metagenomics are currently available for QIIME 2: q2‐shogun (Hillmann et al., 2018) and q2‐metaphlan2 (Truong et al., 2015). They need to be installed separately. In the example below, we demonstrate the use of q2‐shogun, a wrapper for the SHOGUN pipeline (Hillmann et al., 2018).

1 Install QIIME 2 shotgun metagenomics plugins by running:
conda install ‐c bioconda bowtie2
conda install cytoolz
pip install https://github.com/knights‐lab/SHOGUN/archive/master.zip
pip install https://github.com/qiime2/q2‐shogun/archive/master.zip
qiime dev refresh‐cache
2 Download all the required example files from the q2‐shogun repository:
for i in query refseqs taxonomy bt2‐database; do wget https://github.com/qiime2/q2‐shogun/raw/master/q2_shogun/tests/data/$i.qza; done
3 Run shotgun metagenomics pipeline with the following commands:
qiime shogun nobunaga \
‐‐i‐query query.qza \
‐‐i‐reference‐reads refseqs.qza \
‐‐i‐reference‐taxonomy taxonomy.qza \
‐‐i‐database bt2‐database.qza \
‐‐o‐taxa‐table taxatable.qza
  In this example, SHOGUN is called to align query sequences query.qza against a reference sequence database refseqs.qza using the popular short‐sequence aligner Bowtie2 (Langmead & Salzberg, 2012). The query sequences may be demultiplexed or multiplexed data. In the latter case, SHOGUN will automatically stratify alignment results by sample ID. The taxonomy artifact taxonomy.qza defines the mapping of reference sequences to taxonomic lineages. In addition to taxonomy, this artifact could be any hierarchical (semicolon‐delimited) or simple mappings, for example, functional annotations. A Bowtie2 index containing the reference sequence database is necessary for this operation.
  The output file, taxatable.qza, is a feature table in which columns are sample IDs and rows are taxonomic lineages. Starting from this table, we may perform various subsequent analyses in a similar manner to amplicon sequencing data, as detailed above, such as taxonomy plots, alpha and beta diversity analyses, and differential abundance testing.
  If the user wants to prepare a custom reference sequence database from a multi‐FASTA file (e.g., refseqs.fa), it can be done as follows:
*NOTE*: The below sections are presented for demonstration purposes only and are not to be executed unless the file refseqs.fa is first imported by the user.

4 Import the sequences into QIIME 2:
qiime tools import \
‐‐input‐path refseqs.fa \
‐‐type FeatureDate[Sequence] \
‐‐output‐path refseqs.qza
5 Build a Bowtie2 index based on the sequences:
  QIIME 2 is flexible in the types of metagenomic analyses it supports. In addition to calling SHOGUN or MetaPhlAn2 from the QIIME 2 interface, one may perform taxonomic or functional profiling of shotgun metagenomic data separately using any external tool, then import the resulting profile into QIIME 2. BIOM formatted files are supported as input. Questions about other supported formats should be directed to the QIIME 2 Forum, as this will expand over time.

### Source tracking

Source tracking of microbial communities attempts to estimate the relative contributions of a set of host, environmental, and contamination sources to a novel community. QIIME 2 currently offers two methods for microbial source tracking through the external plugins q2‐FEAST (https://github.com/cozygene/FEAST) (Shenhav et al., 2019) and q2‐SourceTracker2 (https://github.com/biota/sourcetracker2) (Knights et al., 2011). FEAST (Fast Expectation‐mAximization microbial Source Tracking) and SourceTracker2 vary in their statistical approach and assumptions for the estimation of source contributions. Therefore, we view method selection here as a personal choice that research teams should make if they do not have a prior hypothesis that one tool addresses directly.

### Compositional data analysis

Feature tables contain magnitudes determined by random sequencing depths that vary dramatically between samples irrespective of the initial microbial load, making the data compositional in nature (Fernandes et al., 2014). Compositional data contain relative information where the abundance of one feature can only be interpreted relative to another.

Numerous normalization methods have been proposed to restore absolute abundances such as rarefaction (Weiss et al., 2017), as well as median (Love, Huber, & Anders, 2014), quantile (Paulson, Stine, Bravo, & Pop, 2013), and constant sum normalization. However, due to erroneous assumptions, these methods cannot control false‐positive rates (Hawinkel, Mattiello, Bijnens, & Thas, 2019; Morton et al., 2017) and contribute to irreproducibility (Fernandes et al., 2014; Gloor, Macklaim, Pawlowsky‐Glahn, & Egozcue, 2017; Gloor, Wu, Pawlowsky‐Glahn, & Egozcue, 2016).

Transformation‐independent and ‐dependent methods developed in the field of compositional data analysis (CoDA) offer an assumption‐free solution (Quinn et al., 2019). Transformation‐dependent methods such as the centered‐ (clr; Aitchison, 1982), isometric‐ (ilr; Egozcue, Pawlowsky‐Glahn, Mateu‐Figueras, & Barceló‐Vidal, 2003), and additive‐ (alr; Aitchison, 1982) log ratio transform the data with regard to a reference. Transformation‐independent methods operate on a single feature or ratios of features (Greenacre, 2019).

CoDA methods rely on logarithms to enforce symmetry in the weighting of relative increases or decreases between features (Aitchison, 1982). The logarithm of zero is undefined, and therefore the non‐trivial task of zero handling is often the first step in CoDA analysis (Silverman, Roche, Mukherjee, & David, 2018). There are many proposed methods (Martín‐Fernández, Barceló‐Vidal, & Pawlowsky‐Glahn, 2003), but QIIME 2 provides two steps to ameliorate the zero problem. First, features that have only a few entries across many samples can be filtered out (https://docs.qiime2.org/2019.10/plugins/available/feature‐table/filter‐features/). Second, a small pseudocount value (often of one) can be added uniformly to the data prior to applying a transform (https://docs.qiime2.org/2019.10/plugins/available/composition/add‐pseudocount/).

After zero handling, multiple CoDA transforms are available in QIIME 2, including clr and ilr on both a hierarchical and phylogenetic basis via gneiss (https://docs.qiime2.org/2019.10/plugins/available/gneiss/) (Morton et al., 2017). Downstream analysis of transformed data is often focused on finding differential features between sample groups. In QIIME 2, both Songbird (https://github.com/biocore/songbird) (Morton et al., 2019) and ALDEx2 (https://github.com/ggloor/q2‐aldex2) (Fernandes et al., 2014) provide supervised differential abundance ranking. QIIME 2 also provides compositional unsupervised dimensionality reduction methods in two forms of Aitchison distance that use different zero‐handling methods (https://docs.qiime2.org/2019.10/plugins/available/diversity/beta/; https://library.qiime2.org/plugins/deicode) (Martino et al., 2019; Pawlowsky‐Glahn, Egozcue, & Tolosana‐Delgado, 2015). Using both supervised and unsupervised CoDA methods, the differential features can be obtained with regard to sample groupings (e.g., armpit versus foot).

After identifying differential features, QIIME 2 also provides methods for transform‐independent analysis using Qurro (https://library.qiime2.org/plugins/qurro; https://doi.org/10.1101/2019.12.17.880047). By taking the log‐ratio between two or the sum of multiple differential features, the sample groupings can be directly visualized.

### Supervised classification and regression methods for predicting sample metadata

Supervised learning (SL) methods predict sample data (e.g., metadata values) as a function of other sample data (e.g., microbiota composition) by training an SL model on training data. Various SL methods can predict either categorical data (a classification problem) or continuous values (a regression problem). SL methods have become increasingly common in microbiome studies to predict sample characteristics (e.g., disease state or location data) or to identify features that are associated with particular characteristics or sample classes (Bokulich, Collins, et al., 2016; Knights et al., 2011; Pasolli, Truong, Malik, Waldron, & Segata, 2016). The ability of many SL methods to perform feature selection—the identification (and ranking) of features associated with particular sample classes or values—is a particularly useful feature of these methods for application in microbiome experiments. The QIIME 2 plugin q2‐sample‐classifier (Bokulich, Dillon, Bolyen, et al., 2018; https://library.qiime2.org/plugins/q2‐sample‐classifier/) contains methods for performing supervised classification/regression and feature selection using microbiome data and metadata.

### Metadata preparation

Metadata are a critical component of a successful study and, unlike other elements such as sequencing quality or completeness of the reference database, metadata are largely under the control of the investigator. Unfortunately, they are often treated as an afterthought, leading to uninterpretable results due to missing information. To ensure a successful data analysis, begin metadata generation at the time of sample collection. Be sure to record all sample attributes that are relevant to your hypotheses, as these attributes are the basis of QIIME 2's visualizations and statistical tests.

Spreadsheets are the most commonly used vehicle for metadata storage and management due to their ubiquity and convenience, but they have well‐known drawbacks. For example, by default Microsoft Excel performs irreversible modification of certain kinds of inputs into dates or floating‐point numbers (Zeeberg et al., 2004), and auto‐completes values based on earlier entries (https://support.office.com/en‐ie/article/turn‐automatic‐completion‐of‐cell‐entries‐on‐or‐off‐0f4aa749‐b927‐4ea7‐adaa‐86f8d4f9fe20). As these modifications are performed silently, without warning to the user, they frequently lead to mangled metadata. Although other spreadsheet programs (such as Google Sheets and LibreOffice) have slightly different defaults, all have “convenience” features that can cause data corruption, so it is critical to learn the default features of your preferred spreadsheet program, follow spreadsheet best‐practices (Broman & Woo, 2018), and actively monitor the validity of your records. Alternatively, generate your metadata file in a dedicated software tool such as ISAcreator (Rocca‐Serra et al., 2010), which provides a structured interface designed to prevent common errors.

Consistency is the key to high‐quality metadata. Much effort has already been put into identifying and standardizing the crucial pieces of metadata for various sorts of studies, so investigate these guidelines before beginning your metadata collection. The Genomic Standards Consortium (GSC) has created the “Minimum Information about any (x) Sequence” (MIxS) and “Minimum Information about a MARKer gene Sequence” (MIMARKS) specifications (Yilmaz et al., 2011) as well as 15 “environmental packages” that extend and refine these standards for samples from environments ranging from air to human skin to waste water. To ease compliance with these standards, the GSC provides checklists outlining the expected inputs, syntax, preferred units, and more for the fields in each standard and package (https://press3.mcs.anl.gov/gensc/mixs/). Many of these fields take values specified by subsets of controlled vocabularies such as the Experimental Factor Ontology (Malone et al., 2010) and the Environment Ontology (Buttigieg et al., 2016). Consider employing a tool such as the stand‐alone ISAconfigurator (Rocca‐Serra et al., 2010) or the Excel‐based QIIMP (The Quick and Intuitive Interactive Metadata Portal; https://qiita.ucsd.edu/iframe/?iframe=qiimp) to identify all the fields necessary for your study type and to enforce the validity of their content.

While creating and maintaining consistent and compliant metadata is not trivial, it is well worth the effort. Not only are standards‐compliant metadata required for submission to a growing number of public databases and journals [e.g., the European Nucleotide Archive (https://www.ebi.ac.uk/training/online/course/ebi‐metagenomics‐portal‐submitting‐metagenomics‐da/what‐are‐metadata‐and‐why‐are‐they‐so‐impo), Qiita (https://qiita.ucsd.edu/static/doc/html/tutorials/prepare‐information‐files.html#required‐fields‐for‐centralized‐qiita), and Microbiome Journal (https://microbiomejournal.biomedcentral.com/submission‐guidelines/preparing‐your‐manuscript/microbiome‐announcement)], but they are also critical to enable future meta‐analyses—both between your own data and others’—and between your own data today and your new data tomorrow! It is much easier to record required information up front than it is to retroactively track this information down when you are working toward a tight paper submission deadline.

## COMMENTARY

### Background Information

Advances in the ease of microbiome data acquisition, due in large part to improved DNA sequencing instruments and standardized protocols, have led to a dramatic increase in the need to analyze the data. However, because so many studies have already been done, it is no longer the best approach to analyze each data set in isolation. Rather, combining the data with what is already known from other studies can have considerable advantages. Therefore, in this article, we show both analysis of an individual dataset and combination with data from other studies. Although we show an example relevant to the human microbiome, we stress that QIIME 2 is useful for analyzing microbiomes from any environment, including soil, seawater, animals, plants, industrial systems, built environments, foods, and a wide range of others that we never anticipated.

QIIME 2 allows rapid meta‐analyses across studies, but these can often be limited due to protocol variation and incomplete or incompatible metadata. Comparing multiple studies directly relies on complete standardized sample metadata, experimental protocols, and bioinformatics analysis. Incomplete metadata with mislabeled or non‐standardized entries severely limits the ability to compare between studies, and leads to spurious conclusions. Experimental protocols that amplify non‐standard variable regions of the 16S rRNA gene, or that use custom sequencing barcodes/adaptors, can also prevent studies from being combined into one analysis. Making the raw data from each study available in public databases, as required by many journals and funding agencies, and using standard methods, can greatly facilitate re‐use and citation of your dataset.

### Critical Parameters

Several points are important to consider before beginning your analysis, and have been reviewed recently to provide best practices for microbiome studies (Allaband et al., 2019; Knight et al., 2018). QIIME 2 tries its best to provide reasonable defaults, but some plugins require considerable biological or subject‐matter knowledge for picking the parameters. Rarefaction provides a good example, because knowledge of which samples cannot be left out of the analysis to obtain biologically meaningful results is needed in order to choose the number of sequences per sample to keep. Similarly, for processing raw sequencing data into a FeatureTable[Frequency] artifact, it is absolutely necessary to know how the samples have been processed, including comprehensive preparation metadata for processing and troubleshooting, as well as the type of barcodes used for multiplexed samples. Metadata must be carefully considered, because the analysis cannot use information that you did not provide. For example, if you want to check whether a parameter such as immune function is correlated with the microbiome, that parameter must actually be measured and included in the metadata table.

Because capabilities in QIIME 2 are rapidly expanding, and because topics such as compositional data analysis are receiving intense focus from the statistics community at present, this document will be continuously updated after publication on the QIIME 2 website (https://qiime2.org/). For questions beyond the scope of this document, we encourage you to use the QIIME 2 forum (https://forum.qiime2.org/), which provides rapid answers to a wide range of questions. We look forward to reading about your applications of QIIME 2 to answer a wide range of compelling questions that touch on the microbiome, whether in the sea, the soil, or the human body!

### Troubleshooting

All actions in QIIME 2 have built‐in help, accessible by including the ‐‐help parameter following any action name, e.g., qiime feature‐table filter‐samples ‐‐help. Additionally, technical support is available through https://forum.qiime2.org, as discussed in the Introduction to this article.

## APPENDIX 1

The QIIME 2 website provides two glossaries, one written for users (see https://docs.qiime2.org/2019.10/glossary/) and one written for developers (see https://dev.qiime2.org/latest/glossary/). The user‐focused glossary is reproduced here as it appeared at the time of publication of this paper. We refer readers to the online version for updated version.

### QIIME 2 User Glossary

**action**: A general term for a *method*, a *visualizer*, or a *pipeline*. Actions are always defined by QIIME 2 *plugins*.

**artifact**: Artifacts are QIIME 2 *results* that are generally considered to represent intermediate data in an analysis, meaning that an artifact is generated by QIIME 2 and intended to be consumed by QIIME 2 (rather than by a human). Artifacts can be generated either by importing data into QIIME 2 or as output from a QIIME 2 *action*. When written to file, artifacts typically have the extension .qza, which stands for “QIIME Zipped Artifact.” Artifacts can be provided as input to QIIME 2 *actions*, loaded with tools such as the QIIME 2 Artifact API for use with Python 3 or qiime2R for use with R, or exported from QIIME 2 for use with other software.

**data provenance**: See *decentralized data provenance*.

**data format**: A *view* of an *artifact* as a file or multiple files stored on disk. QIIME 2 supports many data (or file) formats, and multiple data formats are sometimes available for importing or exporting of QIIME 2 *artifacts* of a given *semantic type*.

**data type**: A *view* of an *artifact* as an in‐memory data representation. Data types are generally only encountered by Artifact API users or *plugin* developers. QIIME 2 supports many data types, and multiple data types are sometimes available for viewing QIIME 2 *artifacts* of a given *semantic type*.

**decentralized data provenance**: Information describing how a QIIME 2 *result* was generated. This will include details on all of the QIIME 2 *actions* that led to the creation of an *artifact*, including the values of all *parameters*, and references to all *inputs* and *results* as *UUIDs*. Data provenance additionally contains the literature citations that are relevant to the generation of a QIIME 2 *result*. Those citations should be included in all published work that derives from a given QIIME 2 *result*. All QIIME 2 *results* contain embedded data provenance which can be visualized with QIIME 2 View (https://view.qiime2.org *)*. Because the data provenance is embedded in the *results* themselves, as opposed to being stored in a centralized database that maintains records on all *results* (for example), QIIME 2's data provenance is described as being decentralized.

**feature**: A unit of observation, such as an operational taxonomic unit, a sequence variant, a gene, or a metabolite. This generic term is used because QIIME 2 can support many different types of features.

**input**: An *artifact* (i.e., non‐primitive) provided to an *action*. For example, table is an input to the filter‐features *action* in the q2‐feature‐table *plugin*.

**method**: A type of QIIME 2 *action* that takes one or more *artifacts* or *parameters* as input, and produces one or more *artifacts* as output. For example, the filter‐features *action* in the q2‐feature‐table *plugin* is a method.

**output**: A *result* generated by running an *action*. For example, filtered‐table is an output from the filter‐features *action* in the q2‐feature‐table *plugin*.

**parameter**: A primitive (i.e., non‐artifact) provided to an *action*. For example, min‐frequency is a parameter to the filter‐features *action* in the q2‐feature‐table *plugin*. See *primitive type*.

**pipeline**: A type of QIIME 2 *action* that typically combines two or more other *actions*. A pipeline takes one or more *artifacts* or *parameters* as input, and produces one or more *results* (*artifacts* and/or *visualizations*) as output. For example, the core‐metrics *action* in the q2‐diversity plugin is a pipeline.

**plugin**: A plugin provides analysis functionality in the form of *actions*. All plugins can be accessed through all interfaces. Plugins can be developed and distributed by anyone. As of this writing, a collection of plugins referred to as the “core distribution” is provided on installation of QIIME 2. Additional plugins can be installed, and the primary resource enabling discovering additional plugins is the QIIME 2 Library (https://library.qiime2.org). Anyone with a QIIME 2 Forum account can share their plugins on the QIIME 2 Library. We plan to phase out the “core distribution” as we move toward distributing all QIIME 2 plugins through the QIIME 2 Library.

**provenance**: See *decentralized data provenance*.

**primitive type**: A type used to define a *parameter* to a QIIME 2 *action*. For example, strings (i.e., text), integers, and booleans (i.e., true or false values) are primitives. Primitives are only used as input to *actions*, and never generated as output by QIIME 2.

**qza**: See *artifact*.

**qzv**: See *visualization*.

**result**: A general term for an *artifact* or a *visualization*.

**sample**: An individual unit of study in an analysis.

**semantic type**: A semantic type describes the meaning of data in QIIME 2. All *results* in QIIME 2 have a single semantic type associated with them, and when importing data into QIIME 2, the user must provide the semantic type of that data. The use of semantic types by QIIME 2 provides an unambiguous way to communicate with others about data, and allows QIIME 2 to reason about data and help users prevent error. An example is helpful for illustrating what semantic types are and how they're used by QIIME 2. QIIME 2 contains two related semantic types, Phylogeny[Rooted] and Phylogeny[Unrooted], which represent rooted and unrooted phylogenetic trees, respectively. Both rooted and unrooted phylogenetic trees can be stored in Newick files, and it is not possible to easily tell if the phylogenetic tree is rooted or not without parsing the file. Some analyses, such as computing UniFrac distances, should be applied only to a rooted phylogenetic tree. By associating a semantic type with a phylogenetic tree artifact, QIIME 2 can determine if the correct type of data is being provided to an *action*, without having to first parse the file (which might be slow, and therefore delay the amount of time before an error can be presented to a user), and then possibly make assumptions based on what is observed. If a user accidentally provides data of a semantic type that is not acceptable for a QIIME 2 *action*, QIIME 2 can quickly detect this mismatch and provide the user with detailed information on the error and how to correct it. Semantic types should not be confused with *data formats*, which define how data are represented on disk. For example, another QIIME 2 semantic type, the FeatureTable[Frequency], can be written to a BIOM‐formatted file or to a tab‐separated text file. By differentiating *data formats* from semantic types, QIIME 2 can support import and export of different file formats based on a user's needs. Semantic types should also not be confused with *data types*. For example, the FeatureTable[Frequency] semantic type could be represented in memory as a biom.Table object or a pandas.DataFrame object, and for different applications, one of these representations might be more useful than the other. Regardless of which in‐memory representation is used, the meaning of the data is the same. By differentiating *data types* and semantic types, QIIME 2 allows developers and users to choose the data structure that is most convenient for them for a given task.

**type**: Type is an ambiguous term, and we therefore try to avoid using it in favor of the more specific terms *semantic type*, *primitive type*, *data format*, or *data type*.

**UUID**: QIIME 2 uses UUIDs, or “Universally Unique Identifiers,” to reference all *results*, and all executions of *actions*. These can be used, for example, to determine that a given *artifact* was generated as output from a specific execution of an *action* using *data provenance*. UUIDs are an unambiguous way to refer to QIIME 2 *results*, because they can never change without invalidating a QIIME 2 *artifact* (unlike file names, for example, which are easy to change and can thus cause errors in tracking *results*).

**view**: A particular representation of an *artifact's* data, for example as a *data format* or *data type*.

**visualizer**: A type of QIIME 2 *action* that takes one or more *artifacts* or *parameters* as input, and produces exactly one *visualization* as output. For example, the summarize *action* in the q2‐feature‐table *plugin* is a visualizer.

**visualization**: Visualizations are QIIME 2 *results* that represent terminal output in an analysis, meaning that they are generated by QIIME 2 and intended to be consumed by a human (as opposed to being consumed by QIIME 2 or other software). Visualizations can only be generated by QIIME 2 *visualizers* or *pipelines*. When written to file, visualizations typically have the extension .qzv, which stands for “QIIME Zipped Visualization.” Visualizations can be viewed with QIIME 2 View (https://view.qiime2.org) on systems that do not have QIIME 2 installed, and QIIME 2 interfaces typically provide their own support for viewing (such as the qiime tools view command available through the QIIME 2 command‐line interface).
